# Narrow therapeutic index drugs: a clinical pharmacological consideration to flecainide

**DOI:** 10.1007/s00228-015-1832-0

**Published:** 2015-04-15

**Authors:** Juan Tamargo, Jean-Yves Le Heuzey, Phillipe Mabo

**Affiliations:** 1 Department of Pharmacology, School of Medicine, University Complutense, 28040 Madrid, Spain; 2 Department of Cardiology, Hôpital Européen Georges Pompidou, Université Paris V René-Descartes, Paris, France; 3 CHU de Rennes, Universite’ Rennes I, Inserm U 642, Rennes, France

**Keywords:** Antiarrhythmic drugs, Flecainide, Generic drugs, Bioequivalence, Narrow therapeutic index, Safety

## Abstract

**Purpose:**

The therapeutic index (TI) is the range of doses at which a medication is effective without unacceptable adverse events. Drugs with a narrow TI (NTIDs) have a narrow window between their effective doses and those at which they produce adverse toxic effects. Generic drugs may be substituted for brand-name drugs provided that they meet the recommended bioequivalence (BE) limits. However, an appropriate range of BE for NTIDs is essential to define due to the potential for ineffectiveness or adverse events. Flecainide is an antiarrhythmic agent that has the potential to be considered an NTID. This review aims to evaluate the literature surrounding guidelines on generic substitution for NTIDs and to evaluate the evidence for flecainide to be considered an NTID.

**Methods:**

A review of recommendations from various regulatory authorities regarding BE and NTIDs, and publications regarding the NTID characteristics of flecainide, was carried out.

**Results:**

Regulatory authorities generally recommend reduced BE limits for NTIDs. Some, but not all, regulatory authorities specify flecainide as an NTID. The literature review demonstrated that flecainide displays NTID characteristics including a steep drug dose–response relationship for safety and efficacy, a need for therapeutic drug monitoring of pharmacokinetic (PK) or pharmacodynamics measures and intra-subject variability in its PK properties.

**Conclusions:**

There is much evidence for flecainide to be considered an NTID based on both preclinical and clinical data. A clear understanding of the potential of proarrhythmic effects or lack of efficacy, careful patient selection and regular monitoring are essential for the safe and rational administration of flecainide.

**Electronic supplementary material:**

The online version of this article (doi:10.1007/s00228-015-1832-0) contains supplementary material, which is available to authorized users.

## Introduction—general considerations on the therapeutic index

The therapeutic index (TI; also known as therapeutic ratio) is a ratio that compares the blood concentration at which a drug causes a therapeutic effect to the amount that causes death (in animal studies) or toxicity (in human studies) [1]. In animal studies, the TI can be calculated as the lethal dose of a drug for 50 % of the population (LD_50_) divided by the minimum effective dose for 50 % of the population (ED_50_), i.e. TI = LD_50_/ED_50_. This ‘academic’ definition of TI is easy to follow in preclinical experiments but opens the door to variable interpretations in clinical practice. In fact, the definition of a therapeutic and/or toxic effect in both animals and humans is highly dependent on the type of therapeutic or toxic effect under consideration.

In clinical practice, the TI is the range of doses at which a medication appeared to be effective in clinical trials for a median of participants without unacceptable adverse effects. For most drugs, this range is wide enough, and the maximum plasma concentration of the drug (*C*_max_) and the area under the plasma concentration–time curve (AUC) achieved when the recommended doses of a drug are prescribed lie sufficiently above the minimum therapeutic concentration and sufficiently below the toxic concentration [2, 3]. Thus, it can be expected that at the recommended prescribed doses, drugs present clinical efficacy with an adequate safety margin.

The range between the ED_50_ and TD_50_ can be considerable, depending on the medication. The larger the TI, the safer the drug is. Conversely, a drug with a narrow TI has generally a steep concentration–response relationship for efficacy, toxicity or both so that there is a narrowly defined range between risk and benefit. It is generally considered that a drug has a good safety profile if its TI exceeds the value of 10. The situation is quite different with the so-called narrow TI drugs (NTIDs), where there is only a very small range of doses at which the drug produces a beneficial effect without causing severe and potentially fatal complications, i.e. small variations in their plasma concentrations can result in an insufficient therapeutic response or appearance of adverse toxic effects. Sometimes, the term ‘critical dose drugs’ is used to refer to drugs in which comparatively small differences in dose or concentration may lead to serious therapeutic failures and/or serious drug reactions [4]. Other terms also used include ‘drugs with a narrow therapeutic window’, ‘narrow therapeutic range’, ‘critical dose drugs’ or ‘narrow therapeutic ratio’.

The US Food and Drug Administration (FDA) defines a drug product as having an NTI when (a) there is less than a twofold difference in median lethal dose (LD_50_) and median effective dose values (ED_50_) or (b) there is less than a twofold difference in the minimum toxic concentrations (MTC) and minimum effective concentrations (MEC) in the blood and (c) safe and effective use of the drug requires careful titration and patient monitoring [4, 5]. A broader definition of an NTID has also been proposed by the FDA in 2011 and is discussed in “Medical considerations on the TI of flecainide” and other antiarrhythmic drugs (AADs).

Because of the small differences between their effective and toxic doses, small changes in the dosage of NTIDs can lead to significant changes in pharmacodynamic (PD) response, and therefore their use should be individualised. This may result in potentially subtherapeutic or toxic effects, particularly in patients with advanced age, with comorbidities or receiving multiple medications.

The concept of an NTI is important for proper use of drugs in medical practice. It is used by clinicians with the subjective but useful notion that drug doses or plasma concentrations associated with a desired therapeutic response are in close proximity to those associated with adverse reactions. The concept of NTI is also used by drug regulatory authorities in the context of drug safety warnings in the summary of drug characteristics of authorised drugs and in the context of guidelines and regulatory standards for bioequivalence (BE) studies (see below).

This review examined the guidance provided from the various national regulatory authorities in relation to BE of NTIDs with a focus on AADs and in particular on flecainide. Additionally, we analysed the possible concerns relating to the substitution of generic drugs for brand name AADs or for other generic medications.

## Bioequivalence studies

In order to be considered a therapeutic equivalent to a brand name drug, a generic drug product must meet pharmaceutical equivalence and BE criteria. Drug products are pharmaceutical equivalents if they contain the same active ingredient(s), have the same dosage form and route of administration and are identical in strength and/or concentration. But generic products may contain different excipients such as colorants, preservatives, lubricants and diluents that might lead to further differences between the two products. It can be argued, however, that by definition excipients are inert substances with no biological activity and generic excipients have to be previously used for approved drugs for which there is evidence that they have not affected the safety or effectiveness [6].

According to the FDA, BE is defined as ‘the absence of a significant difference in the rate and extent to which the active ingredient or active moiety in pharmaceutical equivalents or pharmaceutical alternatives becomes available at the site of drug action (or bioavailability) when administered at the same molar dose under similar conditions in an appropriately designed study’ [7, 8]. In other words, if the innovator and generic drugs are bioequivalent, then they should exhibit equivalent drug concentration–time profiles in the blood [9].

The BE can be established on the basis of the *C*_max_, the time taken for maximum plasma concentration to be reached (*T*_max_), or the AUC, the area under the plasma concentration–time curve from time 0 to the last sampling time [AUC_(0→*t*)_] and AUC_(0→∞)_ (the area under the plasma concentration–time curve from time 0 to infinity for single doses or within a dosing interval at steady state). The usually accepted criterion for concluding that two products are bioequivalent is that the 90 % confidence intervals (CIs) for the ratio between the test and the reference geometric means for AUC and *C*_max_ (determined using log-transformed data) lie within the range of 80.00–125.00 % in the fasting state [7, 9, 10].

The guideline on the investigation of BE of the European Medicine Agency (EMA) indicated that in studies to determine BE after a single dose, the parameters to be analysed are *C*_max_ and AUC_0–*t*_, or, when relevant, AUC_0–72 h_ (in studies with a sampling period of 72 h) [11]. For these parameters, the 90 % CI for the ratio of the test and reference (*T*/*R*) products should be contained within the acceptance interval of 80.00–125.00 %. To be inside the acceptance interval, the lower bound should be ≥80.00 % when rounded to two decimal places, and the upper bound should be ≤125.00 % when rounded to two decimal places.

## Different opinions concerning the BE of NTID

Differences in drug response among patients are common even after the plasma drug concentration has been adjusted to a target value. Because of the very small margin between a safe and lethal dose, NTIDs must be dosed carefully based on plasma concentration, and patients should be monitored closely for any signs of drug toxicity. Due to their potential for ineffective and particularly adverse effects, NTIDs must be kept within an extremely narrow range, which may not be satisfied with current BE standards [12]. Within a population, individual patients show quite substantial variability in response to any one therapy (interindividual variability), and even the same patient can present differences in drug response from dose to dose during the course of drug therapy (intraindividual/within-subject variability) [13–15]. Under these circumstances, measures of population and individual BE are proposed to be more accurate than measures of average BE. Population BE takes into account interindividual variation and is the relevant criterion for a patient being started on a new drug, while individual BE takes into account intraindividual variation and is the relevant criterion for a patient being switched from one formulation to another [13]. Inter- and intraindividual variability are caused by a combination of demographic (gender, body weight or surface area, age and race), gastrointestinal (first-pass metabolism, gastrointestinal pH, motility, blood flow and bacterial flora), environmental (smoking, diet, exposure to pollutants), genetic (associated with variants in drug-metabolising enzymes and/or drug receptors), therapeutic (drug interactions) and physiopathological factors (pregnancy, severity of the disease over time, comorbidities, kidney or hepatic dysfunction) that affect the pharmacokinetics (PK) and/or pharmacodynamics (PD) of a given drug [16, 17]. Monitoring plasma drug concentrations is useful for an NTID when much of the interindividual variability in response is accounted for variability in drug PK, but it has little value when most of the variability is PD. An example of inherited interindividual PK variability is the clearance of flecainide related to CYP2D6 polymorphisms. Thus, caution is warranted when considering generic substitution of drugs that exhibit high interindividual variability or differing PK properties in different patient populations because this variability cannot be addressed by narrowing BE boundaries. Finally, current studies to determine BE are performed in young healthy volunteers who do not take any concurrent medication, and it is assumed that BE in this homogeneous population will equate to all patient populations. Patients with tachyarrhythmias are generally older, present structural heart diseases, are treated with several drugs and present rapid changes in heart rate over time, leading to intraindividual variability in drug PD/PK due to a decrease in cardiac output, volume of distribution and organ perfusion. This population is quite different from that in whom the average BE is performed.

A summary of the BE criteria for general drugs versus NTIDs, with a particular interest on flecainide, is presented in Table 1. In 2010, the Committee for Medicinal Products for Human Use of the EMA indicated that it is not possible to define a set of criteria to categorise drugs as either NTIDs, and cases must be considered individually based on clinical considerations [11]. However, it is recognised that for specific NTIDs, the acceptance interval for AUC should be tightened from the conventional 80.00–125.00 % to 90.00–111.11 % [11, 15, 18]. Where *C*_max_ is of particular importance for safety, efficacy and drug level monitoring, the 90.00–111.11 % acceptance interval should also be applied [11]. Consequently, the tightening acceptance limits for NTID apply both AUC and *C*_max_. Furthermore, the EMA also states: ‘for the purpose of BE requirements, NTIDs may be considered to be those for which there is a risk of clinically relevant difference in efficacy or safety between two products even when the conventional criteria for BE (i.e. 90 % CI for *T*/*R* ratio for AUC and *C*_max_ within 80–125 %) are met’. However, the EMA did not mention whether flecainide is an NTID.

**Table 1:** Different opinions on the BE of NTIDs with a particular interest in flecainide

Agency	BE criteria for general drugs	BE criteria for NTID	Flecainide as an NTID
Foods and Drug Administration (FDA)	80.00–125.00 %	90.00–111.11 %	No
European Medicines Agency (EMA)	80.00–125.00 %	90.00–111.11 %	No
Danish Health and Medicines Authority	80.00–125.00 %	90.00–111.11 %	The agency tightened the BE limits for AADs
Federal Agency for Medicines and Health Products (FAMHP) of Belgium	80.00–125.00 %	90.00–111.11 %	
Health Protection and Food Branch (HPFB) of Canada	80.00–125.00 %	90.00–112.00 %	Flecainide is considered a critical dose drug
New Zealand Medicines and Medical Devices Safety Authority (MEDSAFE)	80.00–125.00 %		Yes
Japanese Institute of Health Sciences (NIHS)	80.00–125.00 %	90.00–111.11 %	Digoxin, disopyramide and quinidine, but not flecainide
Medicines Control Council (MCC) of South Africa	80.00–125.00 %	Tighter limits are considered for NTID	AADs are considered NTID
Therapeutic Goods Administration of Australia (TGA)	80.00–125.00 %	90.00–111.11 %	No list of NTID
Agence Fédérale des Médicaments et des Produits de Santé of Belgium		90.00–111.11 %	Yes
French Agence Nationale de Sécurité des Médicaments (ANSM)			Yes
Agencia Española de Medicamentos y Productos Sanitarios (AEMPS)	80.00–125.00 %	90.00–111.11 %	Yes

*AADs* antiarrhythmic drugs, *BE* bioequivalence, *NTIDs* narrow therapeutic index drugs

The US FDA has not formally designated specific critical dose/NTID or provided a comprehensive list of NTIDs, although warfarin, levothyroxine, carbamazepine, lithium carbonate, digoxin, phenytoin, tacrolimus and theophylline are categorised as NTIDs [19, 20]. The draft guidance on BE studies for flecainide acetate did not mention this drug as an NTID [21]. It should be emphasised that several drugs of the above FDA list of NTIDs (e.g. carbamazepine, lithium) also do not have a mention of their NTI nature in the draft or final guidance document for their BE studies, whereas warfarin and tacrolimus have this mention [22, 23]. However, Laurie Frueh, medical officer from the FDA Office of Pharmaceutical Sciences, in her presentation ‘Interchangeability of critical critical-dose drugs: clinical perspectives’, included antiepileptics, antiarrhythmics, inmmunosupressants and anticoagulants as major drug classes included in this definition [4].

Because of the narrow margin between safe and lethal dosages with NTIDs, some voices from healthcare providers, clinicians, scientists, state regulators, pharmaceutical companies and consumer advocates have expressed concern that bioequivalent generic and brand-name NTI/CD drugs may not be equivalent in their effects on various clinical parameters and proposed that generic substitution should not be applicable for NTID as they deserve special attention and more rigorous BE standards.

The FDA presented the topic of BE for NTIDs in two recent meetings of the Advisory Committee for Pharmaceutical Science and Clinical Pharmacology [4, 19, 24, 25]. In the 2010 meeting, the committee recommended that the agency should develop a list of NTIDs with clear, specialised criteria for including drugs on the list. The list should be made public by the FDA and clearly define the mechanism for addition to the list, the list should be dynamic and constantly monitored and the list should focus on BE issues. Furthermore, the committee made a list of suggestions including ‘replication studies are important’ and ‘the requirements for confidence intervals should perhaps be narrower (90.00–111.11 %) and should include 100 % (or 1.0)’ [19]. In the 2011 meeting, the FDA recommended for NTIDs to conduct a four-way, two-sequence, fully replicated crossover design to assess the within-subject variability of both the test and reference products and to use the reference-scaled average BE approach for the statistical comparison of the relevant PK parameters [19, 26, 27]. The BE limits would change as a function of within-subject variability of the reference product (reference-scaled average BE). If reference variability is ≤10 %, then BE limits are reference-scaled and narrower than 90.00–111.11 %. If reference variability is >10 %, then BE limits are reference-scaled and wider than 90.00–111.11 % but are capped at 80–125 % limits. This proposal encourages development of low within-subject variability formulations [19, 25, 26].

More recently, newly drafted FDA guidance reviews evidence supporting the consideration of warfarin sodium [22] and of tacrolimus [23] as NTIDs; both documents explained the evidence on which the FDA considered warfarin and tacrolimus as NTIDs. The guidance on warfarin recommended a four-way, fully replicated crossover design in order to scale BE limits to the variability of the reference product and compare test and reference product within-subject variability [22]. Furthermore, the guidance described the method for statistical analysis using the reference-scaled average BE approach for NTIDs.

The Canadian Drug Regulatory Agency (Health Canada) in its detailed 2012 Guideline on BE studies [28] did not make a reference to NTI drugs, but they mentioned several exceptions that require modifications of the BE standards. This is the case for the so-called critical dose drugs, defined as those drugs where comparatively small differences in dose or concentration lead to dose- and concentration-dependent, serious therapeutic failures and/or serious adverse drug reactions. For these ‘critical dose drugs’, (a) the 90 % CI of the relative mean AUC of the test to reference formulation should be within 90.00–112.00 % and (b) the 90 % CI of the relative mean *C*_max_ of the test to reference formulation should be 80.00–125.0 % inclusive. These requirements are to be met in both the fasted and fed states. It is important to mention that flecainide is listed in the non-limiting list of ‘critical dose’/NTI drugs. Furthermore, the guideline mentions that due to the nature of these drugs and possibility of serious adverse effects, it may be necessary to conduct BE studies in patients who are already receiving the drug as part of treatment rather than in healthy volunteers, and it is highly recommended that the study group be as homogeneous as possible with respect to predictable sources of variation in drug disposition.

The New Zealand Medicines and Medical Devices Safety Authority (MEDSAFE) considered that NTIDs, such as anticonvulsants, antiarrhythmics, theophylline, warfarin, isotretinoin, cyclosporine and thyroxine, should not be considered as interchangeable drugs [29, 30]. The regulatory guidelines for medicines also stated that tighter limits for permissible differences in bioavailability may be required for medicines that have an NTI, serious dose-related toxicity, a steep dose/effect curve or non-linear PKs within the therapeutic dosage range.

The Therapeutic Goods Administration of Australia (TGA) employs a 90.00–111.11 % BE range instead of the 80–125 % BE range for NTI drugs. This stricter criterion explains why some drugs with an NTI (e.g. warfarin, phenytoin, lithium) do not have generic brands, and switching is not an option in Australia [31].

For the Japanese Institute of Health Sciences (NIHS), the accepted BE acceptance criterion for NTIDs is 90.00–111.11 %. They define NTIDs as those having less than a twofold difference in the minimum toxic concentrations and minimum effective concentrations in the blood and those for which specific drug treatment control fees are approved as remuneration for treatment [32]. The list of 26 NTIDs includes several AADs, including digoxin, disopyramide and quinidine, but not procainamide or flecainide.

The Medicines Control Council (MCC) of South Africa accepts the BE criteria (AUC and *C*_max_ 90 % CI of *T*/*R* ratios should fall within 80–125 %). However, the MCC presents a list of ‘bioproblem’ drugs that includes NTIDs. Furthermore, the substitution guidelines state that ‘unless adequate provision is made for monitoring the patient during the transition period, substitution should not occur when prescribing and dispensing generic medications having a narrow therapeutic range’ [33].

Like the EMA, the Danish Health and Medicines Authority requires tighter acceptance limits, i.e. 90.00–111.11 %, for both AUC and *C*_max_ for drugs with an NTI, including antiarrhythmic agents [34]. The Danish authorities accept that medicinal products containing the NTIDs on their list are therapeutically equivalent because stricter BE criteria were applied, and therefore generic substitution of NTIDs is authorised, with some exceptions.

The Agence Fédérale des Médicaments et des Produits de Santé of Belgium also considered that NTIDs, under certain unspecified conditions, require a narrow (90.00–111.11 %) BE range [35, 36]. They include a list of NTIDs where several AADs, including amiodarone, digoxine, disopyramide, flecainide, propafenone and sotalol (but not quinidine and procainamide), were included. For the Agence, NTIDs are considered to be ‘non-switchable’. The French ANSM (Agence Nationale de Sécurité des Médicaments) has not established an official list of NTIDs. However, flecainide is mentioned as an NTID in the recommendations regarding therapeutic interactions [37].

Finally, the Agencia Española de Medicamentos y Productos Sanitarios of Spain (http://www.aemps.gob.es/) has recently determined that flecainide should be considered an NTID and as such cannot be substituted by pharmacists [38].

Thus, BE guidelines from different representative countries recognise the term NTI (or similar terms), and both the FDA and EMA have discussed the possibility of narrowing the limits of BE from 80.00–125.00 % to even 90.00–111.11 %. As noted, antiarrhythmic agents appeared in the majority of the lists of NTIDs.

## Medical considerations on the TI of flecainide

In general, the concept of TI is not taken into consideration for physicians in daily clinical practice, but the concept becomes obvious to them when adverse reactions occur despite administration of a given drug at the recommended dosing ranges. AADs are considered NTIDs from a clinical point of view for both cardiologists and general practitioners, although the concept of NTI is strongly influenced by the nature of the indication and the presence/absence of underlying heart disease.

It has been considered that NTIDs generally have the following characteristics [19, 20]:

- Steep drug dose–response relationship for both safety and efficacy within the usual dosing range or narrow span between effective drug concentrations and concentrations associated with serious adverse drug reactions. Serious adverse reactions are defined as those which may be persistent, irreversible, slowly reversible, or life-threatening, which could result in in-patient hospitalisation or prolongation of existing hospitalisation, persistent or significant disability or incapacity, or death.
- They are subject to therapeutic drug monitoring based on PK or PD measures to ensure safe and effective use of the drug.
- Small within-subject variability. Otherwise, patients will routinely experience toxicity and lack of efficacy.

## Flecainide as an antiarrhythmic drug

Flecainide is a class IC AAD. In the European Society of Cardiology (ESC) and ACCF/AHA 2012 guidelines for the treatment of atrial fibrillation (AF), flecainide is recommended in the management of patients without structural heart disease (1) for the pharmacological cardioversion of recent-onset AF (recommendation class I, level A), (2) to enhance success of direct current cardioversion and prevent recurrent AF (recommendation class IIa, level B) and (3) for long-term rhythm control (recommendation class I, level A) [39–41] (see Supplementary Table 1 for more details on the clinical indications and contraindications of flecainide as AAD).

## Evidence that flecainide is an NTID

When treating cardiac arrhythmias, where a recurrence might have significant or serious consequences or where the AAD can produce a potentially life-threatening proarrhythmic event, the clinical challenge is to ensure that drug availability at its cardiac receptor site remains constantly within the therapeutic range. Thus, antiarrhythmic therapy represents a challenge for clinicians because potential inefficacy or serious proarrhythmia becomes the issue in a particular patient even though clinical therapeutic BE may hold true for most patients.

The ACCF/AHA (American College of Cardiology Foundation/American Heart Association) 2011 Health Policy Statement on Therapeutic Interchange and Substitution includes a table with examples of NTIDs [42]. The first group of NTIDs was the AADs, and flecainide was included as a representative agent together with digoxin, quinidine, procainamide and disopyramide. Furthermore, as already mentioned, from a regulatory point of view, Health Canada and Spain specifically mention flecainide as an NTID, and other countries, such as Denmark, New Zealand and Belgium, state that AADs have an NTI without specifically mentioning flecainide; Belgium lists flecainide as an NTID in a document referring to prescriptions under non-trade names. Finally, in the treatment of ventricular arrhythmias, flecainide has also been explicitly described as an NTID [3, 43, 44], and two recent comprehensive reviews on the safety profile of flecainide, although they did not mention the term NTI, describe the drug as having such a profile [45, 46].

As already mentioned, flecainide presents the characteristics of an NTID (see Table 2).

**Table 2:** Flecainide presents the pharmacological profile of an NTID

NTI characteristics of flecainide
Steep concentration–response relationships for efficacy, toxicity or both in the usual dosing interval [46, 49, 53, 54, 59, 96]
Dosing generally needs to be titrated according to clinical response [43, 44, 47]
Small differences in dose or blood concentration may lead to serious therapeutic failures and/or adverse drug reactions [43, 49, 54]
There may be a potential for serious clinical consequences in the event of too low or high concentrations [43, 45, 47, 53, 59]
Periodic monitoring of plasma levels is required in patients with severe renal failure or severe hepatic disease [52]
Drug overdose with flecainide is frequently fatal [43, 47, 52, 137]

*NTID* narrow therapeutic index drug

### A steep drug dose–response relationship for both safety and efficacy

Therapeutic trough plasma levels of flecainide associated with greater than 90 % suppression of PVBs range between 200 and 1000 ng/mL [45, 47–51], although cardiac (i.e. conduction defects or bradycardia and ventricular proarrhythmia) or non-cardiovascular adverse reactions may occur in some patients when plasma flecainide levels are ≥700–1000 ng/mL [47, 52–55]. While severe adverse events have been associated at doses twice above the upper limit [56], they may also occur within the therapeutic plasma levels in some patients with cardiovascular diseases [47, 57].

To study the relationship between plasma levels of flecainide and the suppression of ventricular arrhythmias, a decreasing multiple oral dosage regimen (200–50 mg BID) was administered over 12 days in patients with chronic VPBs [47–51]. Maximum plasma levels (413–789 ng/mL) were associated with almost complete (>95 %) suppression of arrhythmias [47–51]. As dose decreased, plasma levels declined to levels below about 230 ng/mL that were associated with a reappearance (<70 % suppression) of arrhythmias. These results suggest that the minimum range of therapeutic plasma levels of flecainide for VPBs is approximately 200–400 ng/mL and that 95 % suppression of VPBs occurs at concentrations up to 800 ng/mL.

In patients with supraventricular tachyarrhythmias treated with oral flecainide (150–300 mg daily), the trough plasma levels vs effect relationship was described as steep [58]. The mean serum flecainide trough concentrations differed significantly between patients with and without palpitations, but the incidence of palpitations was 65 % at serum flecainide concentrations <300 ng/mL and 11 % at ≥300 ng/mL, which indicates that the effective drug plasma concentrations should be maintained at ≥300 ng/mL.

**Fig. 1:**
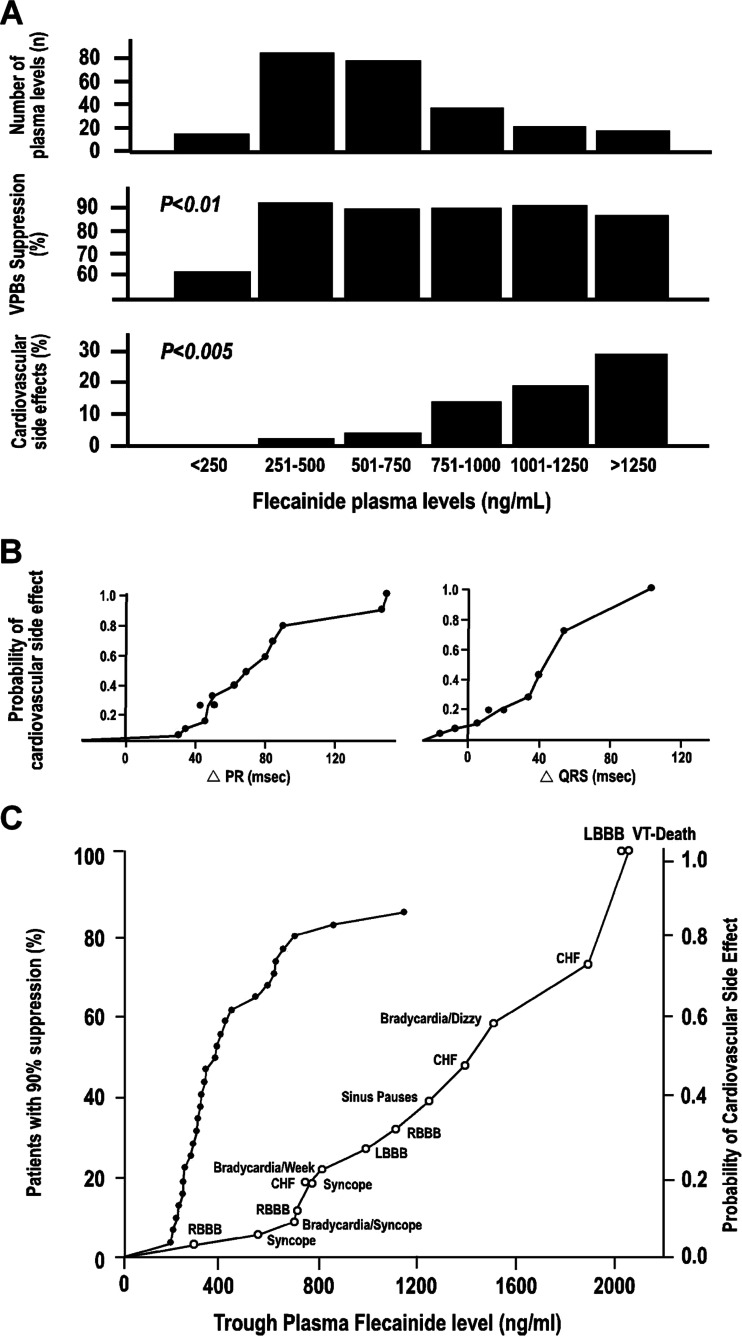
Plasma levels and side effects of flecainide acetate. **a** Flecainide plasma levels, per cent suppression of VPCs from pretreatment and per cent of plasma levels associated with cardiovascular side effects for flecainide plasma levels grouped in 250-ng/mL increments. **b** The probability of cardiovascular side effects compared with the change in ECG intervals from baseline (*n* = 40 for PR and *n* = 36 for QRS interval). Bundle branch block was excluded from analysis for comparison of change in QRS interval with cardiovascular side effects. **c** The probability of cardiovascular side effects occurring is compared with trough plasma flecainide levels by use of the Kaplan–Meier product limit estimator for all 43 patients (*open circles*). The efficacy/plasma concentration curve for 90 % suppression of VEDs is also shown for those 33 patients with available data for both efficacy and flecainide levels (*closed symbols*). Twenty-eight of the 33 patients achieved at least 90 % suppression of VEDs. *RBBB* right bundle branch block, *CHF* congestive heart failure, *LBBB* left bundle branch block, *VPBs* premature ventricular beats, *VT* ventricular trachycardia (taken from Salerno et al.) [57]

Salerno et al. compared the side effects with flecainide trough levels and ECG intervals in patients with ventricular arrhythmias treated with 100–200 mg BID for 34 months [57]. The incidence of adverse cardiovascular effects rose steadily with increasing flecainide plasma levels, but the maximum suppression of VPBs was achieved at flecainide plasma levels of 250–500 ng/mL (Fig. 1). Interestingly, cardiovascular adverse effects related to drug plasma levels but not to the dose of flecainide, so to predict the occurrence of adverse effects, one must monitor some index of the concentration of the drug in plasma. As observed in Fig. 1, the probability of cardiovascular adverse effects begins to rise at a plasma level of approximately 750 ng/mL and reaches 50 % at 1500 ng/mL. The figure also shows the percentage of patients achieving 90 % suppression of VPBs at different drug plasma levels. The correlation between both curves described the therapeutic–toxic window, which is in the range of flecainide plasma levels between 381 ng/mL (at least 50 % probability of efficacy) and 710 ng/mL (less than 10 % probability of cardiovascular side effects). The risk of cardiovascular side effects increases at higher drug plasma levels, and the probability of a cardiovascular event begins to rise sharply at increases of approximately 40 ms in both PR and QRS intervals from baseline. These results confirmed that flecainide dosing is complicated by the steepness of the dose–response for both safety and efficacy.

### The steepness of the dose–response relationship and the risk of proarrhythmia increases in the presence of structural heart disease

**Fig. 2:**
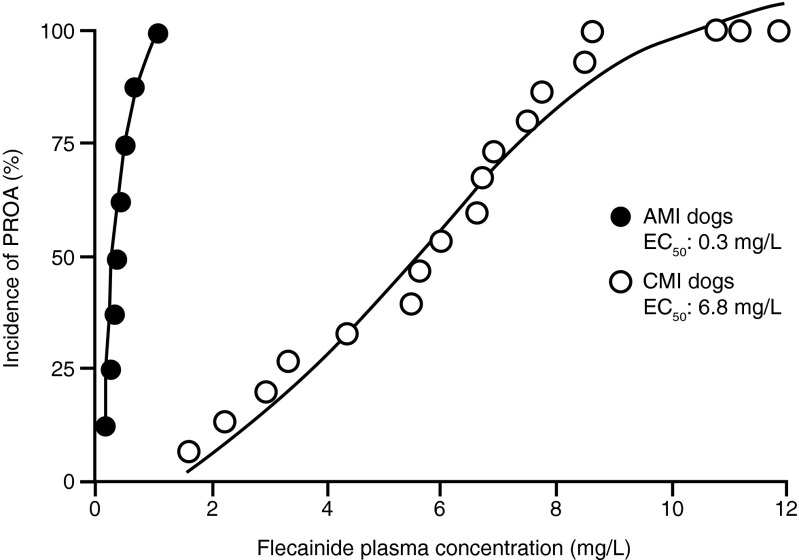
Concentration–response curves for flecainide proarrhythmia in dogs with acute myocardial ischaemia (AMI) or chronic myocardial infarction (CMI) 72 h after coronary artery ligation (taken from Nattel) [60]

The presence of structural heart disease increases the risk of flecainide-induced proarrhythmia. The steepness of the dose–response relationship for drug safety was compared in dogs with 72-h-old healed myocardial ischaemia (MI) and in dogs exposed to acute myocardial ischaemia induced by 10-min coronary occlusions and separated by 30 min of reperfusion [59, 60]. Flecainide-induced proarrhythmia in dogs with healed MI was reported at flecainide plasma concentrations of 0.8–5.6 mg/kg (2–14 μmol/L) [59, 61, 62], which are generally higher than the accepted therapeutic range of 200–700 ng/mL (0.5–1.7 μmol/L). Ventricular proarrhythmia occurred in 31 % of healthy dogs, in 79 % of MI dogs and 55 % of dogs with acute ischaemia. As shown in Fig. 2, flecainide-induced proarrhythmia occurred at therapeutic concentrations in dogs with acute MI (EC_50_ 0.75 μmol/L), concentrations that were 20-fold lower than those producing proarrhythmia in dogs with healed MI (EC_50_ 17 μmol/L) [60]. Furthermore, flecainide-induced proarrhythmia in acute MI generally was ventricular fibrillation (VF), whereas with healed MI proarrhythmia was an inducible sustained VT. These findings strongly suggest an interaction between flecainide and acute myocardial ischaemia, which could explain the excess of deaths in the CAST (Cardiac Arrhythmia Suppression Trial) [63].

In the CAST, the rate of death and non-fatal cardiac arrest increased by 2.3-fold after 10 months of treatment with flecainide in a population with structural heart disease [63]. Interestingly, the excess mortality occurred at non-toxic flecainide doses and was due to sudden cardiac death (SCD) rather than sustained VT. The incidence of SCD was lower when treated with flecainide in combination with a β-blocker than with flecainide alone [64].

As suggested by the authors, acute myocardial ischaemia might facilitate the occurrence of fatal arrhythmias, or the negative inotropic effects of flecainide may have resulted in severe hypoperfusion or increased myocardial oxygen demands during acute ischaemia. Thus, it seems that excess of deaths, myocardial ischaemia and proarrhythmia are interrelated both in experimental models and in post-MI patients. Additionally, the CAST showed that the use of flecainide is not recommended to treat asymptomatic or mildly symptomatic ventricular arrhythmias in patients with LV dysfunction after MI as it increases the mortality risk.

Several studies have demonstrated that flecainide markedly increases the episodes of sustained VT in patients with a history of sustained VT and impaired LV function by almost 20 %, while in patients with stable nonsustained ventricular arrhythmias and preserved LV function, the risk of proarrhythmia appears to be very small [54, 65].

In patients with sustained VT treated with flecainide, 80 % of proarrhythmic events were new or worsened ventricular tachyarrhythmias that occurred within 14 days of the onset of therapy; the remainder was increased frequency of PVCs or new supraventricular arrhythmias. In patients with a history of MI, LV dysfunction and/or an episode of cardiac arrest, the incidence of new or exacerbated ventricular arrhythmias was 13 %, and the incidence of fatal proarrhythmic events was 0.5 % when the dosage was initiated at 200 mg/day and did not exceed 300 mg/day. Using a higher initial dose (400 mg/day), the incidence of proarrhythmic events was 26 %, and the incidence of fatal proarrhythmic events was about 10 %. Patients with sustained VT experienced more frequently new or worsened heart failure (HF) compared with those with supraventricular arrhythmias. Interestingly, the elimination half-life was longer, and plasma clearance was slower in patients with HF [47] as well as in patients with ventricular arrhythmias, even in the absence of HF [43]. Of note is that the co-administration of flecainide with other AADs increased the risk of proarrhythmias, and thus it should be avoided [54, 66].

All of these experimental and clinical studies indicated that the steepness of the dose–response to flecainide as well as the risk of proarrhythmia increases in the presence of structural heart disease, so small changes in drug plasma concentrations can lead to severe proarrhythmia. This explains why flecainide use is contraindicated in patients with AF and a history of structural heart disease involving myocardial ischaemia, hypertrophy and abnormal LV function or congestive HF [39, 40, 67, 68].

### Conduction abnormalities

At therapeutic concentrations, flecainide dose-dependently decreases intracardiac conduction and prolongs the PR, QRS and QT intervals, the effect of the drug being more marked on the His-Purkinje system [45, 46, 51, 55, 69, 70]. The QT prolongation is due to a widening of the QRS complex, while the JT interval and the rate-corrected QT interval (QTc) remain almost unchanged. This explains the rare cases of torsade de pointes caused by flecainide. Flecainide also prolongs atrial, AV nodal and ventricular refractoriness, although the effects on refractoriness are less pronounced than its effects on intracardiac conduction [43–46, 55, 61–63, 69, 71]. Approximately one third of patients may develop new first-degree AV heart block (PR interval ≥0.20 s). Therefore, it is recommended to avoid the combination of flecainide with other AADs as the combination increases the depression of intracardiac conduction and contractility and increases the risk of proarrhythmia. The combination of AADs of different classes implies strict surveillance and control by repeated ECCs, and the combination of AADs of the same class must be avoided.

Even at therapeutic concentrations, flecainide may cause the conversion of AF to a slow atrial flutter at a rate of 200 bpm (1C flutter), and because flecainide does not slow down AV conduction, it may result in a 1:1 AV conduction with a rapid ventricular response [71–73]. Thus, when flecainide is given for prophylaxis against recurrent paroxysmal AF or atrial flutter, AV nodal blocking drugs should be routinely co-administered [40]. Additionally, in some patients, a long asystolic pause may also occur at the time of conversion of AF to sinus rhythm [45, 46, 74]. These adverse effects explain why it is recommended that in patients with AF the first loading oral dose of flecainide should be administered under strict ECG and clinical control in a hospital setting.

### Haemodynamic effects

Both bradycardia and tachycardia have been reported following flecainide administration [43, 54, 55], and an increase in the corrected sinus node recovery time has been described in patients with sinus node dysfunction [53, 55, 56, 66–68]. Multiple doses of oral flecainide had minimal effects on LV ejection fraction in patients with nearly normal ventricular function [43], but flecainide exerts a negative inotropic effect, particularly when given intravenously, and may cause or worsen heart failure in patients with coronary heart disease (CAD), pre-existing HF (New York Heart Association functional class III or IV) or LV dysfunction (LV ejection fraction <30 %) [55, 65, 75–79]. New or worsened HF has been found in 0.4 % of patients (1/22 %) with supraventricular arrhythmias [77] and in 6.3 % of patients (20/317) with PVCs, non-sustained or sustained VT after a mean of 7.9 months of flecainide therapy [67, 68]. However, 25.7 % of patients (78/304) with a history of HF developed worsened HF during a mean duration of 5.4 months of flecainide therapy. This explains why flecainide is contraindicated in patients with congestive HF or LV dysfunction.

### Therapeutic drug monitoring based on PK or PD measures is needed to ensure safe and effective use of the drug

As small changes in flecainide plasma concentrations can lead to severe proarrhythmia, the treatment should start at low dosages that should be increased gradually while monitoring the ECG and/or drug plasma levels to avoid toxic levels (>700–1000 ng/mL) [51, 54, 63]. Nevertheless, the close relationship of drug efficacy and cardiovascular effects with flecainide plasma levels and ECG intervals allows the administration of flecainide at an acceptable safety level [57]. The blockade of cardiac Na^+^ channels slows down intracardiac conduction and prolongs the duration of the QRS complex of the ECG. The QRS widening has been shown to be a surrogate marker for the electrophysiological and therapeutic effect and allows monitoring of cardiac conduction safety [80]. Widening of QRS complex higher than 25 % of the baseline values implies that the dosage must be reduced due to its narrow TI [46]. In case there is a change in the dosage of flecainide or in case the co-prescription of drugs is able to modify cardiac conduction, the patients, mainly those with intracardiac conduction abnormalities, will be carefully monitored by repeated ECGs. In the same way, it is necessary to control the ECG in case of change in the formulation or the presentation of flecainide due to its narrow TI [46].

Flecainide is predominantly excreted in the urine and accumulates in patients with renal failure [81]. Periodic monitoring of plasma levels and ECG is required in patients with severe renal failure or severe hepatic disease. Although the absorption and volume of distribution of flecainide are unaffected in patents with renal impairment, the plasma elimination half-life is significantly prolonged in patients with mild to moderate renal impairment (10–30 h; mean creatinine clearance 37.8 mL/min/1.72 m^2^) and in patients with stage 5 chronic kidney disease (up to 58 h) compared with patients with normal renal function (6–15 h; mean creatinine clearance 106.5 mL/min/1.72 m^2^) [43, 44, 81–83]. Multiple dosing of 100 mg BID in patients with impaired renal function can also result in a longer elimination half-life (45–190 h) and a higher plasma flecainide concentration than single daily dosing [83, 84]. Thus, lower starting and maintenance doses and frequent monitoring of plasma levels and ECG parameters are recommended in patients with moderate to severe renal dysfunction [85]. Monitoring is particularly relevant in patients undergoing dialysis because flecainide is poorly dialysed. Flecainide drug monitoring (plasma levels and ECG changes) are indicated in older patients who present an age-dependent decrease in renal function and multiple comorbidities frequently associated with structural heart diseases (CAD, hypertension, HF, chronic kidney diseases) which result in polypharmacy and increased risk of drug–drug interactions and proarrhythmic effects. Flecainide plasma half-life also increases (19 h) in patients with chronic heart failure as compared with normal subjects [47].

One study evaluated how and to what extent NTIDs (including flecainide), compared with other drugs, were associated with drug-related problems (DRPs) in 827 patients from internal medicine and rheumatology departments from five Norwegian hospitals. The study found that NTIDs were significantly more often associated with DRPs than the non-NTIDs 40 versus 19 % of the times they were used [86]. Three categories of DRPs were significantly more frequently found for NTIDs: non-optimal dose, drug interactions and need for monitoring. These findings demonstrate a particular need for drug surveillance in hospitals to avoid serious consequences of DRP.

### Variability in the PK properties of flecainide

Oral flecainide is rapidly and nearly completely absorbed (bioavailability 90–95 %), reaching *C*_max_ values within 2–3 h and steady state levels within 3–5 days, and it is not altered if it is taken with food. Flecainide is a substrate and an inhibitor of the cytochrome P450 CYP2D6 and presents an apparent plasma half-life that averages approximately 20 h but is quite variable (range 7–30 h) after multiple oral doses in patients with PVBs [45–47]. Flecainide’s disposition kinetics are partly dependent on the CYP2D6 genetic polymorphism [87–92]. Approximately 7–10 % of Caucasians and 1–3 % of Asians are poor metabolisers of drugs metabolised by CYP2D6 [93, 94]. Approximately 30 % (range 10–50 %) of the dose of flecainide is excreted unchanged in urine, mainly by glomerular filtration, but some active tubular secretion may also occur; its metabolites are excreted in urine principally as conjugates [45, 46].

The mean elimination half-life of oral flecainide was found to be 12 h (SD 2.8) and the metabolic clearance 292 (64) mL/min in poor metabolisers compared with 6.9 h (0.9) and 726 (112) mL/min (both *P* < 001), respectively, in rapid metabolisers, while renal clearance did not differ between the groups [92]. However, since the primary route of elimination for flecainide is renal and the proportion of a dose that undergoes hepatic metabolism is small, the role of inter-individual variations in CYP2D6 activity is unlikely to be clinically significant. Even so, a CYP2D6 interaction could become clinically relevant in patients with renal dysfunction. Recently, it has been demonstrated that the CYP2D6 genotype is a determinant factor of age-related decline in flecainide clearance [88]. The metabolic clearance was decreased age-dependently in a curvilinear fashion greater than 60 years for heterozygous extensive metabolisers (het-EMs) and greater than 55 years for intermediate metabolisers (IMs) and poor metabolisers (PMs). The reduction in metabolic clearance in elderly (70 years) patients compared with middle-aged (52 years) patients was different among the CYP2D6 genotype groups: 22.1 and 49.5 % in CYP2D6 IMs and PMs, respectively, with no change in homozygous extensive metabolisers (hom-EMs). Because an 11.4 % reduction in estimated glomerular filtration rate in elderly patients compared with middle-aged patients corresponded to a 6.1 % decline in flecainide clearance, the age-related decline in flecainide clearance was 6.1 % in hom-EMs, 16.3 % in het-EMs and 28.9 % in IMs/PMs groups.

Although these differences will not usually be of clinical importance, they may have a significant impact in patients with chronic kidney disease and/or treated with other drugs that could affect the hepatic metabolism of flecainide. In fact, in patients with PM/PM CYP2D6 genotype and renal insufficiency, flecainide has been associated with death [95]. Furthermore, the PK of flecainide differs between subjects with the *CYP2D6* wild-type (*1 or *2) allele and the *10 allele in Japanese patients with supraventricular tachyarrhythmia using routine therapeutic drug monitoring data, which confirms that these differences may be clinically relevant in the East Asian population [90]. PK interaction with paroxetine, a potent inhibitor of CYP2D6, has been reported in healthy volunteers who had the CYP2D6*10 allele, which is frequent in Asians with clinical consequences (flecainide-induced delirium) [93, 96].

The flecainide elimination rate from plasma can be markedly slower in patients with significant hepatic impairment. In these patients, flecainide should be used only when the potential benefits clearly outweigh the risks, and if used, close monitoring (ECG and drug plasma levels) is required. Patients with HF had slightly longer half-lives (19 vs 14 h in healthy subjects) and lower mean plasma clearance (8.1 vs 10.2 mL/min/kg) [55].

## Safety profile of flecainide

Despite all of these conditions, when used in appropriately selected patients, flecainide presents a good safety profile as demonstrated by more than 25 years’ of cumulative experience in Europe and the USA [45, 46, 55].

Because of the concern about the safety of flecainide following the publication of the CAST trial, the database of 236 patients with supraventricular arrhythmias treated in the USA with flecainide was obtained from 3M Pharmaceuticals and compared with that in a research arrhythmic clinic, the Duke population (154 patients) [97]. The 6-year survival functions of these two populations, estimated by Kaplan–Meier technique, did not differ significantly. This finding demonstrated that the increase in mortality found in patients with ventricular arrhythmias in the CAST trial cannot be extrapolated to patients with supraventricular arrhythmias.

Recent clinical trials have reported a good tolerability profile for flecainide in groups of appropriately selected patients, i.e. when patients with HF, LV dysfunction, cardiac hypertrophy and/or CAD were excluded in line with current treatment guidelines [39–41, 98].

When used in patients with supraventricular arrhythmias without detectable structural heart disease and when drug plasma levels are maintained within the therapeutic range (0.2–1 μg/mL), flecainide has been shown to have a good safety profile, and it may contribute to suppression of AF- or SVT-related symptoms. In a meta-analysis of 122 prospective studies conducted in 4811 patients (mean age 55 ± 13 years, 60 % male) with supraventricular arrhythmias and no significant signs of LV damage, with a mean exposure time of 241 ± 224 days, the rate of proarrhythmic events was significantly lower in flecainide (mean dose 216 ± 65 mg/day) compared with placebo recipients (2.7 vs 4.8 %, *P* < 0.001) [99]. In this analysis, there was no significant difference between the flecainide and control groups in the rate of SCD or total mortality, although there were 120 proarrhythmic episodes observed in 120 flecainide-treated patients and 88 in control patients (*P* < 0.001). The yearly all-cause mortality rate was estimated to be 0.397 per 100 person-years. Of the cardiac deaths, all but two occurred in patients with CAD. Compared with controls, flecainide was associated with a lower incidence of proarrhythmic episodes (2.7 vs 4.8 %), angina symptoms (1 vs 1.3 %), hypotension (0.8 vs 1.3 %), diarrhoea (0.7 vs 2.8 %), headache (2.0 vs 2.9 %) and nausea (1.6 vs 1.8 %), and less than 5 % of patients receiving flecainide discontinued treatment due to adverse effects [99]. These results confirmed the importance of selecting patients without structural heart disease before initiating the treatment with flecainide.

In a study evaluating the cardiac safety of the 200-mg controlled-release formulation of flecainide in the prevention of paroxysmal AF, the mean maximum QRS increase from baseline was 11.4 %, and only four patients had a maximum QRS value >100 ms under treatment [100]. The most frequent drug-related proarrhythmic effects were bradycardia (13.2 %), ventricular extrasystoles (10.6 %), AV block (4.0 %), supra-ventricular tachycardia (2.2 %), bundle branch block (1.8 %) and AF (1.3 %). New or worsened arrhythmias occurred in 1 % of patients with PSVT and in 0.4 % of patients with paroxysmal AF/flutter treated with oral flecainide, while 10.5 % of patients with chronic AF developed ventricular tachyarrhythmias; therefore, flecainide is not recommended in these patients.

A nationwide study enrolled 141,500 patients admitted with AF in Denmark from 1995 to 2004 [101]. In this unselected population, the use of AADs, including flecainide, was not associated with an increased risk of death. The annualised mortality rate in the flecainide cohort (*n* = 3356; mean dosage of 205.6 mg) was 2.54 per year per 100 person-years, which compares favourably with the corresponding rates for patients treated with propafenone (4.25), sotalol (5.29) and amiodarone (7.42). This study confirmed that in selected patients with AF, treatment with flecainide was not associated with increased risk of death. Despite the promising results, this study was limited by its retrospective non-randomised nature.

However, other studies have demonstrated that flecainide is effective in preventing AF recurrences, but even in this population flecainide still carries a clinically significant risk of potentially severe adverse events at therapeutic doses [52, 102–105]. Flecainide-induced ventricular proarrhythmia manifests as monomorphic wide QRS tachycardia or as polymorphic VT or VF. Factors associated with ventricular proarrhythmia risk include decreased LV function, ventricular scar tissue, too high a dose and/or rapid dose increases. Premonitory signs on the surface ECG include excessive increases in QRS duration [73, 106, 107].

One study evaluated the cardiac safety of 200-mg controlled-release formulation of flecainide in the prevention of paroxysmal AF in 227 patients. After 24 weeks of treatment, the incidence of paroxysmal AF decreased from 28.6 to 11 %, 131 patients (71.8 %) had a QRS duration increase of <15 % of the patients and 34 patients (18.8 %) had a QRS increase ≥25 % [100]. Bradycardia (13.2 %) and ventricular extrasystoles (10.6 %) were the most frequently identified proarrhythmic effects, while atrio-ventricular block (4.0 %), supraventricular tachycardia (2.2 %), bundle branch block (1.8 %) and AF (1.3 %) were the most frequent drug-related cardiac adverse events. Almost 7 % of patients discontinued therapy due to cardiac adverse events.

In another study, 112 patients with paroxysmal (51 %) or persistent (49 %) AF (mean age 60 ± 11 years) were treated with flecainide (mean dose 203 ± 43 mg/day) [108]. Eight deaths were reported during a mean follow-up of 3.4 years. Three deaths were classified as SCD and occurred at 9 days, 20 and 63 months after the start of treatment. Six patients discontinued the treatment due to proarrhythmia. Thus, the incidence of SCD or proarrhythmia was 8 %, resulting in an annual incidence of almost 2.5 %. Compared to the general population, the standardised mortality ratios were 1.6 (95 % CI 0.68–3.09) for all-cause mortality and 4.2 (95 % CI 1.53–9.06) for death from cardiovascular disease during flecainide exposure. These harmful effects were not prevented by careful evaluation during drug initiation and follow-up or by treatment with AV-blocking agents, which confirm the proarrhythmic risk of flecainide.

In previous studies, one randomised controlled trial that compared flecainide with placebo [109] and five that compared flecainide with an alternative anti-arrhythmic treatment in AF populations [110–114] describe eight deaths in the AF population receiving flecainide (three because of non-cardiac causes, four because of structural heart disease; no information available in the remaining case). Death occurred between 1 day and 2 months after flecainide treatment initiation. No deaths were reported in the 313 patients in another six studies with a median flecainide exposure time of 12 months [115–120]. In 568 patients with paroxysmal atrial flutter/AF treated with oral flecainide, VT appeared in 0.4 % (2/568) of these patients. Conversely, in 19 patients in the literature with chronic AF, two (10.5 %) experienced VT or VF. Therefore, flecainide is not recommended in this population [67, 68]. In 225 patients with supraventricular arrhythmia (108 with paroxysmal supraventricular tachycardia and 117 with paroxysmal AF), there were nine (4 %) proarrhythmic events; seven episodes were exacerbations of supraventricular arrhythmias and two were ventricular arrhythmias, including one fatal case of VT/VF and one wide complex VT, both in patients with paroxysmal AF and known CAD [67, 68].

A recent analysis of 56 studies comprising 20,771 patients who recovered sinus rhythm after AF assessed the effect of long-term treatment with AADs on death, stroke and embolism, adverse effects, pro-arrhythmia and recurrence of AF. Several class IA (disopyramide, quinidine), IC (flecainide, propafenone) and III (amiodarone, dofetilide, dronedarone, sotalol) AADs significantly reduced the recurrence of AF (OR 0.19 to 0.70, number needed to treat 3 to 16). However, all of these AADs increased withdrawals due to adverse effects, and all but amiodarone, dronedarone and propafenone increased pro-arrhythmia, and some of them (disopyramide, quinidine and sotalol) may increase mortality [121].

In another direct meta-analysis, all of these AADs were shown to be efficacious at reducing AF recurrence. Treatment withdrawals specifically due to adverse effects were significantly increased for all AADs, and all drugs were associated with an increased risk of proarrhythmia compared with placebo. Based on a limited number of patients, flecainide showed an increase in the risk of serious adverse events compared with placebo (OR 10.36, 95 % CI 1.26–58.24) [102, 122].

Flecainide can organise and slow down the rate of AF, converting it to atrial flutter, which in some patients (3.5–5 %) with a particularly slow atrial rate may result in 1:1 AV conduction with a rapid ventricular response [72, 73]. This complication is a risk that has limited the prescription of flecainide in patients with supraventricular arrhythmias and is more likely to occur in the presence of adrenergic stimulation. Drugs inducing the prolongation of AV conduction time nodes, β-blockers, calcium antagonists and possibly digoxin, have been proposed as useful concomitant medication during treatment with class IC antiarrhythmics.

### Risk of ventricular proarrhythmia

The major concern with starting antiarrhythmic therapy for AF is the potential to induce ventricular proarrhythmia. Estimating the risk for ventricular arrhythmia is particularly important in deciding whether to initiate AAD therapy under close in-patient monitoring or on an outpatient basis. Flecainide, like other AADs, can induce the occurrence of a more severe arrhythmia than the arrhythmia for which it has been prescribed. It can also increase the heart rate of a previously diagnosed arrhythmia or worsen the severity of symptoms. A spontaneous variation of the arrhythmia due to the patient’s condition may be difficult to distinguish from a worsening due to the drug itself. The occurrence of more frequent or polymorphic premature ventricular complexes implies that the treatment must be stopped. In case of heart failure history, due to the negative inotropic effect of the drug, flecainide will be prescribed under a strict surveillance of cardiac function in patients with a history of symptoms suggestive of heart failure [39, 40, 45, 46, 55].

In one study, flecainide was administered to 152 patients (100–400 mg orally to 46; 2 mg/kg intravenously to 106) over a period of 22 months [66]. Fourteen patients (8.1 %) developed proarrhythmic effects. Five patients developed new ventricular tachyarrhythmias— three VF and two VT (three of these patients had pre-existing ventricular arrhythmias); two of these patients were taking other AADs, and proarrhythmic effects occurred with both normal and high flecainide concentrations. Two patients developed supraventricular tachyarrhythmias, and seven experienced bradyarrhythmias. In one patient, flecainide resulted in an increase of atrial flutter cycle length, which resulted in the development of 1:1 atrioventricular conduction rate and, overall, a faster ventricular rate.

An initial survey performed in 544 patients with ventricular arrhythmias by the manufacturer following the distribution of an Adverse Effects Questionnaire found a proarrhythmic response in 44 (8 %); 33 of these patients developed new or worsened ventricular tachyarrhythmias. Of the 44 patients who had a proarrhythmic response, 30 (11.8 %) were among the 254 patients with haemodynamically significant VT or VF and advanced structural heart diseases, whereas the incidence of ventricular proarrhythmia decreased to 4.2 % in patients with chronic non-sustained ventricular arrhythmias [54]. However, the population enrolled in this survey included patients with decreased LV function, previous MI, history of sustained VT or concomitant treatment with other class I AADs, i.e. patients in whom the administration of flecainide is presently contraindicated. Indeed ventricular proarrhythmia seems to be rare when there is preserved LV function and in the absence of other predisposing factors such as electrolyte disturbances [45, 46, 72]. A systematic review of seven trials determined that the incidence of ventricular arrhythmias in flecainide-treated patients was <3 % [123].

New or exacerbated ventricular arrhythmias occurred in 7 % of patients with PVBs, non-sustained or sustained VT. Among patients treated for sustained VT (who frequently had previous history of LV dysfunction, HF or MI), the incidence of proarrhythmic events was 13 % when the treatment was initiated at the dose of 200 mg/day and after titration did not exceed 300 mg/day in most patients but increased to 26 % when the starting dose was higher (400 mg/day). Because of the high frequency of proarrhythmic events, flecainide is contraindicated in patients with sustained VT and underlying heart disease, and drug therapy in suitable patients should be started in the hospital.

The risk of proarrhythmia and other serious adverse effects can be minimised (Table 3) by keeping strict adherence to prescribing guidelines, with a better understanding of the pharmacology of the drugs prescribed, limiting the number of drugs prescribed, starting the treatment at low doses that will be increased on the basis of the patient’s response and comorbidities and performing regular monitoring of the ECG and, if possible, of the drug plasma levels. As previously mentioned, even the self-administration of a single oral dose of flecainide shortly after the onset of symptomatic AF (‘pill-in-the-pocket’) can be administered to terminate persistent AF outside the hospital only after treatment has been previously proven safe in the hospital [40]. Importantly, current methods of determining BE and therapeutic equivalence do not account for PK variation and do not offer information regarding the intra-patient variability, the differences in PKs that happen within the same patient from dose to dose during the course of drug therapy [124].

**Table 3:** Recommendations to minimise the proarrhythmic effects of flecainide

Recommendations
Keep strict adherence to prescribing guidelines
Avoid the use of flecainide in patients with structural heart disease
A better understanding of the pharmacology of the drugs prescribed
It will allow to identify possible drug interactions
Limit the number of drugs prescribed
Avoid the concomitant use of other antiarrhythmic drugs
Start the treatment at low doses that will be increased on the bases of patient’s response and comorbidities
Increase dose after reaching steady-state levels (within 3–6 days)
Therapeutic drug monitoring (ECG, drug plasma levels) is recommended when making drug adjustments
Particularly in the elderly and in patients with hepatic and/or renal dysfunction
Monitor drug plasma levels to avoid toxic levels (>1000 ng/mL)
Check the efficiency and in particular the safety of the drug after the transition from an in-hospital to the ambulatory setting
Pill-in-the-pocket approach: only when flecainide has been previously proved safe in hospital and has a specific approval

*ECG* electrocardiogram

## Drug interactions

The effects of drugs and other conditions on flecainide plasma concentrations are summarised in Supplementary Table 2.

## Generic substitution of brand-name AADs with a narrow TI

When a patient is treated for a benign, non-life-threatening disease with a drug that has a wide therapeutic window, generic substitution (i.e. switching between a branded drug and its therapeutically equivalent generic version) is not a clinical problem for many brand-name drugs. There might be, however, serious concerns about generic substitution for NTIDs in which a relatively small change in drug plasma levels can result in marked changes in PD response. For AADs such as flecainide which show a steep dose–response relationship, small changes in drug plasma levels can lead to successful treatment, recurrence of the arrhythmia, a proarrhythmic event or even death [2, 3]. Thus, it seems reasonable to avoid formulation substitution from the brand-name drug to a generic drug, from a generic drug to another generic drug or from a generic drug to a brand-name drug, when the possible recurrence of the arrhythmia due to a lower drug plasma concentration or when an increase in drug plasma levels due to a PK interaction can lead to a serious event for the patient. It seems reasonable to avoid substitution if recurrence of the arrhythmia being treated could be fatal when the tissue level of the drug falls or if an increase in serum plasma levels of the drug can also be associated with a life-threatening outcome, such as a ventricular proarrhythmia, especially if warning signs might not appear first.

For ethical reasons, the conventional BE studies between generics and brand-name drugs are usually single-dose, two-treatment crossover studies performed in a small number of young healthy volunteers to minimise interindividual PK variability. In this homogeneous population, PK endpoints can be fairly consistent. However, the BE parameters change with age or in the presence of a disease and flecainide is usually prescribed in older patients (average age of patients with AF is over 65 years) who present smaller body size (particularly women), lower rates of hepatic metabolism and impaired renal excretion and several other comorbidities that sensitise them to the proarrhythmic effects of flecainide and most of them (more than 80 %) take at least one drug every day increasing the appearance of drug interactions [125]. Indeed flecainide can interact with other antiarrhythmics and other drugs widely prescribed in patients with frequent cardiac arrhythmias, such as propranolol, diltiazem, verapamil or digoxin [45, 46] or with non-cardiovascular drugs (Supplementary Table 2).

The question is whether the BE studies performed in small numbers of fasting, healthy, normal volunteers, often homogeneous in characteristics such as age and gender, can be extrapolated into therapeutic equivalence patients with a variety of disease conditions. Furthermore, although a difference of 20 % between brand name and generic products is used to define BE, it should be mentioned that BE relates to the mean of the data for the study population, so values for individual subjects may lie outside the BE intervals, even though the mean ratios (and CIs) are within the limits [2, 3]. Because of the steep dose–response curve of flecainide and the interindividual differences in drug metabolism and half-life, the 20 % difference between products commonly used to define BE can lead to small variations in drug exposure, leading to reduced antiarrhythmic efficacy or adverse effects on patient outcomes. Thus, it is not a surprise that some claim that under these circumstances it would be desirable for BE tests to be carried out on patients.

Some sparse evidence indicated that formulation substitution involving AADs (quinidine, procainamide, disopyramide, amiodarone) can be associated with clinical equivalence, inequivalence [2, 3] and sometimes with adverse clinical consequences [2, 3, 126–132]. Indeed a trend toward differences in *C*_max_, *t*_max_ and AUC were reported for two proprietary brands of sustained-release procainamide [127], and adverse clinical consequences, including arrhythmia recurrence (associated with lower plasma concentrations) or proarrhythmia (associated with higher plasma concentrations) have been reported in association with generic substitution of procainamide [128, 129], quinidine [133], amiodarone [2, 3] and sotalol [3]. In most cases, arrhythmia control was re-established when the patients were switched back to the trade-name drug [2, 3]. These results can be attributed partly to differences in drug absorption as BE studies were performed in healthy volunteers while these older studies enrolled some post-myocardial patients in whom gastrointestinal absorption is altered [134].

A survey of 130 expert electrophysiologists analysed their experience with formulation substitution using AADs [124]. They provided 54 cases of recurrent tachyarrhythmia: 21 with a class IA AADs, one with metoprolol and 32 with amiodarone following generic substitution. Among these recurrences, there were three deaths due to VF, and more may have been seen in the absence of implantable cardioverter defibrillator backup, thus raising serious concerns about both AADs. In one of the patients, there was a marked decrease in serum amiodarone concentrations following a Pacerone substitution for Cordarone (from 1.4 to 0.2 ng/mL). Another patient presented a recurrence of AF with a rapid ventricular response to a quinidine formulation substitution, which simultaneously resulted in a decline in the digoxin blood concentration. In this case, formulation substitution resulted in a change in serum and tissue levels of one drug which, in turn, led to changes in the concentrations of a second agent if the PK of this second agent is directly influenced by the amount of the first agent [2, 3].

No study is available regarding the comparison of the TI of flecainide under its two formulations: immediate release (IR) and controlled release (CR). The effect of single- and repeated-dose PKs and electrocardiographic effects (QRS duration) of both formulations was performed in 24 healthy subjects to examine the influence of CYP2D6 activity [87]. The CR formulation produced a lower *C*_max_ and delayed time to reach *C*_max_; however, trough flecainide plasma concentration at steady state was bioequivalent for both formulations, and maximum and minimum QRS increases were not significantly different for either the IR or the CR form of flecainide after administration of both single and repeated doses. Mean QRS duration during a dosing interval at steady state correlated with mean plasma concentration for both forms, and CYP2D6 polymorphism did not appear to influence flecainide disposition kinetics or electrocardiographic effects at steady state. Another comparative study in 48 patients with paroxysmal AF between flecainide IR (100 mg BID) and CR (200 mg OD) using QRS duration as the primary endpoint demonstrated ECG PD equivalence between both formulations. However, there was a marked difference in circadian QRS variation pattern, with peaks and troughs appearing with the IR formulation but not under the CR formulation [135]. This result is consistent with a greater occurrence of frequency-dependent QRS variations over the 24-h period with the IR compared with the CR formulation.

Nevertheless, this evidence can be considered as anecdotal case reports, although it can also be taken as hypothesis-generating evidence for future comparisons between brand name and generic drugs or even among different generics. However, such comparative trials will not be performed in the near future due to inadequate post-marketing funds available from the budgets of the innovator drugs and from the generic manufacturers, lack of administrative support on both sides of the Atlantic and perhaps concerns about marketing risks, depending upon the outcome [2, 3].

Thus, the clinician should be aware for possible changes in the PD effects when a brand name is substituted by a generic or when switching between different generic products, particularly with drugs for which safe and effective use requires careful titration and patient monitoring such as AADs. Thus, generic substitution may not be advisable or even allowable for NTIDs [55]. In fact, the American Medical Association strongly recommends that therapeutic interchange in patients with chronic diseases who are stabilised on a drug-therapy regimen be discouraged and indicated that, especially for drugs with a narrow therapeutic range, therapeutic drug concentration or PD monitoring is necessary to assure the desired clinical response [136]. If formulation substitution is undertaken, it is strongly recommended to employ rigorous monitoring of drug levels and/or ECG or other PD markers. Finally, any prescriber of NTIDs must be clear on the differences between brand name and generic drugs, as well as any reported differences between generics [11].

## Conclusions

Flecainide is an NTID not only for clinicians but also for several regulatory agencies as well as for the American College of Cardiology Foundation Clinical Quality Committee [42]. Preclinical and clinical studies found that flecainide presents a steep dose–response curve that is even accentuated in the presence of structural heart disease (particularly in the ischaemic myocardium), evidence of potentially serious clinical consequences when high flecainide plasma concentrations are reached (although serious adverse effects have been reported even at ‘therapeutic’ concentrations) and the need for careful monitoring of the ECG and plasma drug levels under several circumstances such as in patients with renal or hepatic insufficiency. Cardiac adverse effects observed in patients with cardiac arrhythmias include potentially severe and sometimes life-threatening proarrhythmic events, particularly in patients with structural heart disease (i.e. ischaemic heart disease or HF). All of these characteristics confirm that flecainide can be considered as an NTID.

However, flecainide is a drug of choice for the management of patients with paroxysmal and persistent AF in carefully selected groups of patients according to the ESC guidelines because it is effective and has a good safety profile and a low risk of ventricular proarrhythmia. To ensure that the benefits of treatment outweigh the risks, a clear understanding of the potential to produce proarrhythmic effects, careful selection of patients and regular monitoring of the patient is essential for the safe and rational administration of flecainide.

## Electronic supplementary material

Below is the link to the electronic supplementary material.

ESM 1 (DOC 82 kb)

ESM 2 (DOC 146 kb)
