# Supplementary material for: Narrow therapeutic index drugs: a clinical pharmacological consideration to flecainide
Source: Eur J Clin Pharmacol. 2015 Apr 15;71(5):549–67. doi: 10.1007/s00228-015-1832-0 (PMC4412688; doi:10.1007/s00228-015-1832-0)
Supplement: Supplementary file 1 — (DOC 82 kb) [file 228_2015_1832_MOESM1_ESM.doc]

Supplementary Table 1. Key clinical indications and contraindications of flecainide as antiarrhythmic agent [1–5]

| **Indications** |
| --- |
| 1. In patients without structural [heart disease](http://www.rxlist.com/script/main/art.asp?articlekey=31193), flecainide is indicated for the prevention of:   - Paroxysmal supraventricular tachycardias (PSVT), including AV nodal re-entrant [tachycardia](http://www.rxlist.com/script/main/art.asp?articlekey=5698), AV re-entrant tachycardia and other supraventricular tachycardias of unspecified mechanism associated with disabling symptoms - Paroxysmal AF associated with disabling symptoms - Documented [ventricular arrhythmias](http://www.rxlist.com/script/main/art.asp?articlekey=5978), such as sustained VT, that in the judgment of the physician are life-threatening and the benefit of treatment overweighs proarrhythmic risks   2. Flecainide (‘pill-in-the-pocket’) in addition to a beta blocker or nondihydropyridine calcium channel antagonist to terminate AF out of hospital in selected patients once this treatment has been observed to be safe in a monitored setting |
| **Contraindications/not recommended** |
| Contraindications:   - Structural heart disease - Pre-existing second- or third-degree AV block, or right bundle branch block when associated with a left hemiblock (bifascicular block), unless a temporary or implanted pacemaker is present to ensure an adequate ventricular rate if complete heart block occurs - Presence of cardiogenic shock, cardiac failure, previous MI, haemodynamically significant valvular heart disease, long-standing untreated AF or known hypersensitivity to the drug or Brugada syndrome   Not recommended:   - Long-term (permanent) AF - Less severe [ventricular](http://www.rxlist.com/script/main/art.asp?articlekey=26787) arrhythmias even in [symptomatic](http://www.rxlist.com/script/main/art.asp?articlekey=20424) patients - With extreme caution in patients with sick sinus syndrome due to the risk of [sinus bradycardia](http://www.rxlist.com/script/main/art.asp?articlekey=19707), sinus pause or sinus arrest - With caution in patients with existing poor thresholds or non-programmable pacemakers, unless suitable pacing rescue is available |
| *ADD* antiarrhythmic drug; *AF* atrial fibrillation; *AV* atrioventricular; *LV* left ventricular; *MI* myocardial infarction; *PVB* premature ventricular beats; *VT* ventricular tachycardia  Notes: use of intravenous flecainide should be initiated in the hospital; use with extreme caution in patients with sick sinus syndrome due to the risk of [sinus bradycardia](http://www.rxlist.com/script/main/art.asp?articlekey=19707), sinus pause or sinus arrest |

**References**

1. Fuster V, Rydén LE, Cannom DS, et al. (2006) ACC/AHA/ESC 2006 Guidelines for the Management of Patients with Atrial Fibrillation: a report of the American College of Cardiology/American Heart Association Task Force on Practice Guidelines and the European Society of Cardiology Committee for Practice Guidelines (Writing Committee to Revise the 2001 Guidelines for the Management of Patients With Atrial Fibrillation): developed in collaboration with the European Heart Rhythm Association and the Heart Rhythm Society. Circulation 114:e257–354. doi: 10.1161/CIRCULATIONAHA.106.177292
2. Camm AJ, Lip GYH, De Caterina R, et al. (2012) 2012 focused update of the ESC Guidelines for the management of atrial fibrillation: an update of the 2010 ESC Guidelines for the management of atrial fibrillation. Developed with the special contribution of the European Heart Rhythm Association. Eur Heart J 33:2719–2747. doi: 10.1093/eurheartj/ehs253
3. Anderson JL, Halperin JL, Albert NM, et al. (2013) Management of patients with atrial fibrillation (compilation of 2006 ACCF/AHA/ESC and 2011 ACCF/AHA/HRS recommendations): a report of the American College of Cardiology/American Heart Association Task Force on Practice Guidelines. J Am Coll Cardiol 61:1935–1944. doi: 10.1016/j.jacc.2013.02.001
4. European Heart Rhythm Association, Heart Rhythm Society, Zipes DP, et al. (2006) ACC/AHA/ESC 2006 guidelines for management of patients with ventricular arrhythmias and the prevention of sudden cardiac death: a report of the American College of Cardiology/American Heart Association Task Force and the European Society of Cardiology Committee for Practice Guidelines (Writing Committee to Develop Guidelines for Management of Patients With Ventricular Arrhythmias and the Prevention of Sudden Cardiac Death). J Am Coll Cardiol 48:e247–346. doi: 10.1016/j.jacc.2006.07.010
5. Tamargo J, Capucci A, Mabo P (2012) Safety of flecainide. Drug Saf 35:273–89. doi: 10.2165/11599950-000000000-00000
