# Supplementary material for: Narrow therapeutic index drugs: a clinical pharmacological consideration to flecainide
Source: Eur J Clin Pharmacol. 2015 Apr 15;71(5):549–67. doi: 10.1007/s00228-015-1832-0 (PMC4412688; doi:10.1007/s00228-015-1832-0)
Supplement: Supplementary file 2 — (DOC 146 kb) [file 228_2015_1832_MOESM2_ESM.doc]

# Supplementary material

Supplementary Table 2. Effects of drugs and conditions on flecainide plasma concentrations

| **Drug** | **Pharmacodynamic** | **Pharmacokinetic** | **Implications** |
| --- | --- | --- | --- |
| Amiodarone [1–4] |  | Inhibits CYP2D6, decreases the renal clearance of flecainide and increases FPC by twofold or more | This combination is not recommended. If used, reduce the dose of flecainide by 50%. Monitor patients closely for adverse reactions |
| AADs: class I and III [3–7] | Higher risk of bradycardia, depression of intracardiac conduction and contractility and proarrhythmia. |  | Concomitant use of flecainide and other antiarrhythmic agents should be avoided |
| Beta-adrenergic blockers [8] |  |  | In the CAST trial, sudden death rate was lower in patients treated with flecainide plus β-blocker than in patients treated with flecainide alone |
| Boceprevir [9] |  | Increases FPC | Increases the risk of proarrhythmia. Monitor ECG and FPC |
| Bupropion [10] |  | May increase FPC | Should be given with caution |
| Calcium channel blockers: diltiazem, verapamil [3,4,6,11] | Added negative inotropic and impair AV nodal conduction |  | Monitor the ECG |
| CYP2D6 inducers (carbamazepine, phenytoin, phenobarbital, primidone) [3,4,12,13] |  | Increase (30%) the rate of flecainide elimination, especially in extensive metabolisers | Increase the dose of flecainide |
| CYP2D6 inhibitors:  Cimetidine [14]  Darifenacin [15]  Lumefantrine [16]  Protease inhibitors (amprenavir, darunavir, fosamprenavir, indinavir, lopinavir, ritonavir, saquinavir, tipranavir) [12,13]  Quinidine [17,18]  Selective serotonin reuptake inhibitors (citalopram, fluoxetine, paroxetine, sertraline) [19–21]  [Serotonin-norepinephrine reuptake inhibitor](http://en.wikipedia.org/wiki/Serotonin-norepinephrine_reuptake_inhibitor)s (duloxetine, venlafaxine) [22]  Terbinafine [23] |  | Increase FPC and half-life, especially in extensive metabolisers  Quinidine decreases total and nonrenal clearance of flecainide (24 and 28%) | Reduce the dose of flecainide to avoid potential proarrhythmic effects.  These combinations should be approached with caution. Consider to avoid the combination of these agents with flecainide |
| Digoxin [24–26] |  | Digoxin plasma concentrations increased (15–19%) 6 h after its co-administration with flecainide | Close monitoring of the ECG and serum digoxin concentrations is recommended during combination therapy |
| Diuretics: thiazide and loop diuretics [4,27] | Produce hyponatraemia and hypokalaemia that might increase flecainide-induced cardiotoxicity | Increase FPC | Correct electrolyte abnormalities before flecainide administration |
| Etravirine [28] |  | Decreases FPC | Monitor the effects of flecainide |
| Hyperkalaemia [6] | Increases flecainide-induced conduction disturbances |  | Hyperkalaemia should be corrected before flecainide administration |
| Propranolol [12,13,24] | Added negative inotropic and AV nodal depressant effects | Increased the plasma levels of both drugs (20–30%) | Uncertain clinical meaning. It can be necessary to reduce the dose of flecainide |
| QTc-prolonging drugs^a^ [3,4,12,13] | QT prolongation and increased risk of proarrhythmia |  | Avoid the co-administration when possible |
| Tobacco [29] |  | Stimulates cytochrome P450 activity and increases the clearance of flecainide | Smokers may need larger doses of flecainide to achieve the same therapeutic effects |
| Urine acidifiers or alkalinisers [6,30,31] |  | Urine alkaliniation (sodium bicarbonate) decreases urinary excretion of flecainide.  Urine acidification (ammonium chloride) may increase flecainide excretion | The clinical significance of these findings remains to be determined. |
| CAD [12,13,32,33] | Increased proarrhythmia and mortality in patients with myocardial infartion | They present a decrease in renal function | Careful titration and monitoring (ECG and PFC). Flecainide should not be used in patients with recent myocardial infarction |
| Elderly [3–6] |  | Flecainide elimination from plasma is somewhat slower | Monitor the ECG during dosage adjustment |
| Heart failure or LV dysfunction [3,4,12,13,34–37] | Flecainide has negative inotropic effects. Increases the risk of heart failure and proarrhythmia | Increases the half-life (19 hours) and decreases drug clearance | The initial dosage should be >100 mg bid. Careful titration and monitoring (ECG and PFC). Optimise heart failure treatment |
| Hemodialysis | Removes ~1% of an oral dose as unchanged flecainide |  | Not an effective for removing flecainide from the body |
| Hepatic impairment [3,4,6,38] |  | Slow elimination rate of flecainide | Avoid flecainide use unless the potential benefits clearly outweigh the risks |
| Intracardiac conduction defects [3–7,33,39-43] | Flecainide slows intracardiac conduction |  | Reduce the dose when PR intervals ≥0.30 sec or the widening of the QRS complex is higher than 25% of the baseline values  Avoid in patients with second or third degree AV block, or right bundle branch block associated with a left hemiblock, unless a pacemaker is implanted |
| Pregnancy [12,13] |  |  | Flecainide should be used only if the potential benefit justifies the potential risk to the foetus (Pregnancy Category C) |
| Proarrhythmia [3–6, 39–44] | The risk increases in patients with structural heart disease (coronary artery disease, hypertrophy, heart failure) |  | Follow the recommended dosage |
| Renal impairment [3,4,45–48] |  | Excretion of unchanged drug in urine is reduced and the plasma half-life of flecainide is prolonged. In PMs higher FPC may be anticipated, which may result in proarrhythmic effects | crCl ≤35 mL/min/1.73 m^2^: initial dosage 50-100 mg QD. ECG and PFC monitoring is required to guide dosage adjustments. crCl 35-60 mL/min/1.73 m^2^: initial dosage 50-100 mg QD |
| Sick Sinus Syndrome [6,39,49] | Flecainide may cause sinus bradycardia, sinus pause, or sinus arrest |  | Flecainide should be used only with extreme caution |

^a^Available from: https://www.crediblemeds.org/ (alfuzosin, artemether, ciprofloxacin, antiarrhythmic drugs, citalopram, clozapine, dronederone, gadobutrol, lumefantrine, macrolide antibiotics (erythromycin), [mesoridazine](http://www.drugbank.ca/drugs/DB00933), mizolastine, nilotinib, pimozide, tacrolimus, tetrabenazine, thioridazine, toremifene, trimipramine, voriconazole, vorinostat, ziprasidone, [zuclopenthixol](http://www.drugbank.ca/drugs/DB01624))

*AADs* antiarrhythmic drugs, *crCl* creatinine clearance, *CAD* coronary artery disease, *FPC* flecainide plasma concentrations, *LV* left ventricular, *PMs* poor metabolisers

# Supplementary table references

1. [Shea P](http://www.ncbi.nlm.nih.gov/pubmed/?term=Shea P%5BAuthor%5D&cauthor=true&cauthor_uid=3958371), [Lal R](http://www.ncbi.nlm.nih.gov/pubmed/?term=Lal R%5BAuthor%5D&cauthor=true&cauthor_uid=3958371), [Kim SS](http://www.ncbi.nlm.nih.gov/pubmed/?term=Kim SS%5BAuthor%5D&cauthor=true&cauthor_uid=3958371), [Schechtman K](http://www.ncbi.nlm.nih.gov/pubmed/?term=Schechtman K%5BAuthor%5D&cauthor=true&cauthor_uid=3958371), [Ruffy R](http://www.ncbi.nlm.nih.gov/pubmed/?term=Ruffy R%5BAuthor%5D&cauthor=true&cauthor_uid=3958371) (1986) Flecainide and amiodarone interaction. [J Am Coll Cardiol](http://www.ncbi.nlm.nih.gov/pubmed/?term=Shea+P+1986) 7:1127–1130
2. Funk-Brentano C, [Becquemont L](http://www.ncbi.nlm.nih.gov/pubmed/?term=Becquemont L%5BAuthor%5D&cauthor=true&cauthor_uid=8143391), [Kroemer HK](http://www.ncbi.nlm.nih.gov/pubmed/?term=Kroemer HK%5BAuthor%5D&cauthor=true&cauthor_uid=8143391), [Bühl K](http://www.ncbi.nlm.nih.gov/pubmed/?term=Bühl K%5BAuthor%5D&cauthor=true&cauthor_uid=8143391), [Knebel NG](http://www.ncbi.nlm.nih.gov/pubmed/?term=Knebel NG%5BAuthor%5D&cauthor=true&cauthor_uid=8143391), [Eichelbaum M](http://www.ncbi.nlm.nih.gov/pubmed/?term=Eichelbaum M%5BAuthor%5D&cauthor=true&cauthor_uid=8143391), [Jaillon P](http://www.ncbi.nlm.nih.gov/pubmed/?term=Jaillon P%5BAuthor%5D&cauthor=true&cauthor_uid=8143391) (1994) Variable disposition kinetics and electrocardiographic effects of flecainide during repeated dosing in humans: contribution of genetic factors, dose-dependent clearance, and interaction with amiodarone. [Clin Pharmacol Ther](http://www.ncbi.nlm.nih.gov/pubmed/8143391) 55:256–269
3. Aliot E, Capucci A, Crijns HJ, Goette A, Tamargo J (2011) Twenty-five years in the making: flecainide is safe and effective for the management of atrial fibrillation. Europace 13:161–173
4. Tamargo J, Capucci A, Mabo P (2012) [Safety of flecainide.](http://www.ncbi.nlm.nih.gov/pubmed/22435343) Drug Saf 35:273–289
5. Morganroth J, Horowitz LN (1984) Flecainide: its proarrhythmic effect and expected changes on the surface electrocardiogram. Am J Cardiol 53:89B–94B
6. Holmes B, Heel RC (1985) Flecainide. A preliminary review of its pharmacodynamic properties and therapeutic efficacy. Drugs 29:1–33
7. [Nathan AW](http://www.ncbi.nlm.nih.gov/pubmed?term=Nathan AW%5BAuthor%5D&cauthor=true&cauthor_uid=4006779), [Hellestrand KJ](http://www.ncbi.nlm.nih.gov/pubmed?term=Hellestrand KJ%5BAuthor%5D&cauthor=true&cauthor_uid=4006779), [Bexton RS](http://www.ncbi.nlm.nih.gov/pubmed?term=Bexton RS%5BAuthor%5D&cauthor=true&cauthor_uid=4006779), [Spurrell RA](http://www.ncbi.nlm.nih.gov/pubmed?term=Spurrell RA%5BAuthor%5D&cauthor=true&cauthor_uid=4006779), [Camm AJ](http://www.ncbi.nlm.nih.gov/pubmed?term=Camm AJ%5BAuthor%5D&cauthor=true&cauthor_uid=4006779) (1985) The proarrhythmic effects of flecainide. [Drugs](http://www.ncbi.nlm.nih.gov/pubmed/4006779#%23) 29(suppl 4):45–53
8. Kennedy HL, Brooks MM, Barker AH, Bergstrand R, Huther ML, Beanlands DS Bigger JT, Goldstein S (1994) Beta-blocker therapy in the Cardiac Arrhythmia Suppression Trial. CAST Investigators. Am J Cardiol 74:674–680
9. Schering Corporation (2011) Prescribing information for Victrelis® (boceprevir) capsules. <http://www.accessdata.fda.gov/drugsatfda_docs/label/2011/202258lbl.pdf>. Accessed January 2015
10. GlaxoSmithKline (2014) Prescribing information for Wellbutrin® (bupropion hydrochloride) tablets. <https://www.gsksource.com/gskprm/htdocs/documents/WELLBUTRIN-TABLETS-PI-MG.pdf>. Accessed January 2015
11. Holtzman JL, Finley D, Mottonen L, Berry DA, Ekholm BP, Kvam DC, McQuinn RL, Miller AM (1989) The pharmacodynamic and pharmacokinetic interaction between single doses of flecainide acetate and verapamil: effects on cardiac function and drug clearance. Clin Pharmacol Ther 46:26–32
12. Amneal Pharrmaceuticals (2013) Flecainide prescribing information. <http://www.drugs.com/pro/flecainide.html>. Accessed August 2014
13. Meda Pharmaceuticals Ltd (2013) Flecainide acetate - Summary of product characteristics (UK). <https://www.medicines.org.uk/emc/medicine/3905>. Accessed August 2014
14. Tjandra-Maga TB, van Hecken A, van Melle P, Verbesselt R, de Schepper PJ (1986) [Altered pharmacokinetics of oral flecainide by cimetidine.](http://www.ncbi.nlm.nih.gov/pubmed/3741720) Br J Clin Pharmacol 22:108–110
15. Novartis (2004) Prescribing information for Enablex® (darifenacin) extended-release tablets. <http://www.accessdata.fda.gov/drugsatfda_docs/label/2012/021513s010lbl.pdf>. Accessed January 2015
16. Novartis (2009) Prescribing information for Coartem® (artemether/lumefantrine) tablets. <https://www.pharma.us.novartis.com/product/pi/pdf/coartem.pdf>. Accessed January 2015
17. Munafo A, Buclin T, Tuto D, Biollaz J. (1992) The effect of a low dose of quinidine on the disposition of flecainide in healthy volunteers. Eur J Clin Pharmacol. 43:441–443
18. Birgersdotter UM, Wong W, Turgeon J, Roden DM (1992) [Stereoselective genetically-determined interaction between chronic flecainide and quinidine in patients with arrhythmias.](http://www.ncbi.nlm.nih.gov/pubmed/1576047) Br J Clin Pharmacol 33:275–280
19. [Lim KS](http://www.ncbi.nlm.nih.gov/pubmed?term=Lim KS%5BAuthor%5D&cauthor=true&cauthor_uid=18754843), [Cho JY](http://www.ncbi.nlm.nih.gov/pubmed?term=Cho JY%5BAuthor%5D&cauthor=true&cauthor_uid=18754843), [Jang IJ](http://www.ncbi.nlm.nih.gov/pubmed?term=Jang IJ%5BAuthor%5D&cauthor=true&cauthor_uid=18754843), [Kim BH](http://www.ncbi.nlm.nih.gov/pubmed?term=Kim BH%5BAuthor%5D&cauthor=true&cauthor_uid=18754843), [Kim J](http://www.ncbi.nlm.nih.gov/pubmed?term=Kim J%5BAuthor%5D&cauthor=true&cauthor_uid=18754843), [Jeon JY](http://www.ncbi.nlm.nih.gov/pubmed?term=Jeon JY%5BAuthor%5D&cauthor=true&cauthor_uid=18754843), [Tae YM](http://www.ncbi.nlm.nih.gov/pubmed?term=Tae YM%5BAuthor%5D&cauthor=true&cauthor_uid=18754843), [Yi S](http://www.ncbi.nlm.nih.gov/pubmed?term=Yi S%5BAuthor%5D&cauthor=true&cauthor_uid=18754843), [Eum S](http://www.ncbi.nlm.nih.gov/pubmed?term=Eum S%5BAuthor%5D&cauthor=true&cauthor_uid=18754843), [Shin SG](http://www.ncbi.nlm.nih.gov/pubmed?term=Shin SG%5BAuthor%5D&cauthor=true&cauthor_uid=18754843), [Yu KS](http://www.ncbi.nlm.nih.gov/pubmed?term=Yu KS%5BAuthor%5D&cauthor=true&cauthor_uid=18754843) (2008) Pharmacokinetic interaction of flecainide and paroxetine in relation to the CYP2D6*10 allele in healthy Korean subjects. Br J Clin Pharmacol 66:660–666
20. Tsao YY, Gugger JJ (2009) Delirium in a patient with toxic flecainide plasma concentrations: the role of a pharmacokinetic drug interaction with paroxetine. Ann Pharmacother 43:1366–1369
21. Nemeroff CB, DeVane CL, Pollock BG (1996) Newer antidepressants and the cytochrome P450 system. Am J Psychiatry 153:311–320
22. Lilly USA. Prescribing information for Cymbalta® (Duloxetine Delayed-Release Capsules) for oral use. <http://pi.lilly.com/us/cymbalta-pi.pdf>. Accessed January 2015
23. Novartis (1992) Prescribing information for Lamisil® (terbinafine hydrochloride) oral granules. <http://www.pharma.us.novartis.com/product/pi/pdf/Lamisil_Oral_Granules.pdf>. Accessed January 2015
24. Lewis GP, Holtzman JL (1984) Interaction of flecainide with digoxin and propranolol. Am J Cardiol 53:52B–57B
25. Tjandramaga TB, Verbesselt R, Van Hecken A, Mullie A, De Schepper PJ (1982) Oral digoxin pharmacokinetics during multiple-dose flecainide treatment. Arch Int Pharmacodyn Ther 260:302–303
26. [Weeks CE](http://www.ncbi.nlm.nih.gov/pubmed?term=Weeks CE%5BAuthor%5D&cauthor=true&cauthor_uid=3950050), [Conard GJ](http://www.ncbi.nlm.nih.gov/pubmed?term=Conard GJ%5BAuthor%5D&cauthor=true&cauthor_uid=3950050), [Kvam DC](http://www.ncbi.nlm.nih.gov/pubmed?term=Kvam DC%5BAuthor%5D&cauthor=true&cauthor_uid=3950050), [Fox JM](http://www.ncbi.nlm.nih.gov/pubmed?term=Fox JM%5BAuthor%5D&cauthor=true&cauthor_uid=3950050), [Chang SF](http://www.ncbi.nlm.nih.gov/pubmed?term=Chang SF%5BAuthor%5D&cauthor=true&cauthor_uid=3950050), [Paone RP](http://www.ncbi.nlm.nih.gov/pubmed?term=Paone RP%5BAuthor%5D&cauthor=true&cauthor_uid=3950050), [Lewis GP](http://www.ncbi.nlm.nih.gov/pubmed?term=Lewis GP%5BAuthor%5D&cauthor=true&cauthor_uid=3950050) (1986) The effect of flecainide acetate, a new antiarrhythmic, on plasma digoxin levels. J Clin Pharmacol 26:27–31
27. [Ohki R](http://www.ncbi.nlm.nih.gov/pubmed?term=Ohki R%5BAuthor%5D&cauthor=true&cauthor_uid=11227957), [Takahashi M](http://www.ncbi.nlm.nih.gov/pubmed?term=Takahashi M%5BAuthor%5D&cauthor=true&cauthor_uid=11227957), [Mizuno O](http://www.ncbi.nlm.nih.gov/pubmed?term=Mizuno O%5BAuthor%5D&cauthor=true&cauthor_uid=11227957), [Fujikawa H](http://www.ncbi.nlm.nih.gov/pubmed?term=Fujikawa H%5BAuthor%5D&cauthor=true&cauthor_uid=11227957), [Mitsuhashi T](http://www.ncbi.nlm.nih.gov/pubmed?term=Mitsuhashi T%5BAuthor%5D&cauthor=true&cauthor_uid=11227957), [Katsuki T](http://www.ncbi.nlm.nih.gov/pubmed?term=Katsuki T%5BAuthor%5D&cauthor=true&cauthor_uid=11227957), [Ikeda U](http://www.ncbi.nlm.nih.gov/pubmed?term=Ikeda U%5BAuthor%5D&cauthor=true&cauthor_uid=11227957), [Shimada K](http://www.ncbi.nlm.nih.gov/pubmed?term=Shimada K%5BAuthor%5D&cauthor=true&cauthor_uid=11227957) (2001) Torsades de pointes ventricular tachycardia induced by mosapride and flecainide in the presence of hypokalemia. Pacing Clin Electrophysiol 24:119–121
28. Janssen Cilag (2013) Prescribing information for Intelence® (etravirine) tablets for oral use. <http://www.intelence.com/shared/product/intelence/prescribing-information.pdf>. Accessed January 2015
29. [Holtzman JL](http://www.ncbi.nlm.nih.gov/pubmed?term=Holtzman JL%5BAuthor%5D&cauthor=true&cauthor_uid=2501055), [Weeks CE](http://www.ncbi.nlm.nih.gov/pubmed?term=Weeks CE%5BAuthor%5D&cauthor=true&cauthor_uid=2501055), [Kvam DC](http://www.ncbi.nlm.nih.gov/pubmed?term=Kvam DC%5BAuthor%5D&cauthor=true&cauthor_uid=2501055), [Berry DA](http://www.ncbi.nlm.nih.gov/pubmed?term=Berry DA%5BAuthor%5D&cauthor=true&cauthor_uid=2501055), [Mottonen L](http://www.ncbi.nlm.nih.gov/pubmed?term=Mottonen L%5BAuthor%5D&cauthor=true&cauthor_uid=2501055), [Ekholm BP](http://www.ncbi.nlm.nih.gov/pubmed?term=Ekholm BP%5BAuthor%5D&cauthor=true&cauthor_uid=2501055), [Chang SF](http://www.ncbi.nlm.nih.gov/pubmed?term=Chang SF%5BAuthor%5D&cauthor=true&cauthor_uid=2501055), [Conard GJ](http://www.ncbi.nlm.nih.gov/pubmed?term=Conard GJ%5BAuthor%5D&cauthor=true&cauthor_uid=2501055) (1989) Identification of drug interactions by meta-analysis of premarketing trials: the effect of smoking on the pharmacokinetics and dosage requirements for flecainide acetate. Clin Pharmacol Ther 46:1–8
30. Muhiddin KA, Johnston A, Turner P (1984) [The influence of urinary pH on flecainide excretion and its serum pharmacokinetics.](http://www.ncbi.nlm.nih.gov/pubmed/6326790) Br J Clin Pharmacol 17:447–451
31. Johnston A, Warrington S, Turner P (1985) [Flecainide pharmacokinetics in healthy volunteers: the influence of urinary pH.](http://www.ncbi.nlm.nih.gov/pubmed/4074602) Br J Clin Pharmacol. 20:333–338
32. Fuster V, Rydén LE, Cannom DS, Crijns HJ, Curtis AB, Ellenbogen KA, Halperin JL, Le Heuzey JY, Kay GN, Lowe JE, Olsson SB, Prystowsky EN, Tamargo JL, Wann S, Smith SC Jr, Jacobs AK, Adams CD, Anderson JL, Antman EM, Halperin JL, Hunt SA, Nishimura R, Ornato JP, Page RL, Riegel B, Priori SG, Blanc JJ, Budaj A, Camm AJ, Dean V, Deckers JW, Despres C, Dickstein K, Lekakis J, McGregor K, Metra M, Morais J, Osterspey A, Tamargo JL, Zamorano JL; American College of Cardiology/American Heart Association Task Force on Practice Guidelines; European Society of Cardiology Committee for Practice Guidelines; European Heart Rhythm Association; Heart Rhythm Society (2006) ACC/AHA/ESC 2006 guidelines for the management of patients with atrial fibrillation: a report of the American College of Cardiology/American Heart Association Task Force on Practice Guidelines and the European Society of Cardiology Committee for Practice Guidelines (Writing Committee to Revise the 2001 Guidelines for the Management of Patients With Atrial Fibrillation). Circulation 114:e25–e354
33. Zipes DP, Camm AJ, Borggrefe M, Buxton AE, Chaitman B, Fromer M, Gregoratos G, Klein G, Moss AJ, Myerburg RJ, Priori SG, Quinones MA, Roden DM, Silka MJ, Tracy C, Smith SC Jr, Jacobs AK, Adams CD, Antman EM, Anderson JL, Hunt SA, Halperin JL, Nishimura R, Ornato JP, Page RL, Riegel B, Priori SG, Blanc JJ, Budaj A, Camm AJ, Dean V, Deckers JW, Despres C, Dickstein K, Lekakis J, McGregor K, Metra M, Morais J, Osterspey A, Tamargo JL, Zamorano JL; American College of Cardiology; American Heart Association Task Force; European Society of Cardiology Committee for Practice Guidelines (2006) [ACC/AHA/ESC 2006 guidelines for management of patients with ventricular arrhythmias and the prevention of sudden cardiac death: a report of the American College of Cardiology/American Heart Association Task Force and the European Society of Cardiology Committee for Practice Guidelines (Writing Committee to Develop Guidelines for Management of Patients With Ventricular Arrhythmias and the Prevention of Sudden Cardiac Death).](http://www.ncbi.nlm.nih.gov/pubmed/16949478) J Am Coll Cardiol 48:e247–e346
34. Serruys PW, Vanhaleweyk G, Van Den Brand M, Verdouw P, Lubsen J, Hugenholtz PG (1983) The haemodynamic effect of intravenous flecainide acetate in patients with coronary artery disease. Br J Clin Pharmacol 16:51–59
35. Muhiddin KA, Turner P, Blackett A (1985) Effect of flecainide on cardiac output. Clin Pharmacol Ther 37:260–263
36. Legrand V, Materne P, Vandormael M, Collignon P, Kulbertus HE (1985) Comparative haemodynamic effects of intravenous flecainide in patients with and without heart failure and with and without beta-blocker therapy. Eur Heart J 6:664–671
37. Hellestrand KJ, Bexton RS, Nathan AW, Spurrell RA, Camm AJ (1982) Acute electrophysiological effects of flecainide acetate on cardiac conduction and refractoriness in man. Br Heart J 48:140–148
38. Conard GJ, Ober RE (1984) Metabolism of flecainide. Am J Cardiol 53:41B–51B
39. Roden DM, Woosley RL (1986) Drug therapy. Flecainide. N Engl J Med 315:36–41
40. Estes NA III, Garan H, Ruskin JN (1984) Electrophysiologic properties of flecainide acetate. Am J Cardiol 53:26B–29B
41. Anderson JL, Stewart JR, Crevey BJ (1984) A proposal for the clinical use of flecainide. Am J Cardiol 53:112B–119B
42. [Boriani G](http://www.ncbi.nlm.nih.gov/pubmed?term=Boriani G%5BAuthor%5D&cauthor=true&cauthor_uid=8150547), [Capucci A](http://www.ncbi.nlm.nih.gov/pubmed?term=Capucci A%5BAuthor%5D&cauthor=true&cauthor_uid=8150547), [Strocchi E](http://www.ncbi.nlm.nih.gov/pubmed?term=Strocchi E%5BAuthor%5D&cauthor=true&cauthor_uid=8150547), [Calliva R](http://www.ncbi.nlm.nih.gov/pubmed?term=Calliva R%5BAuthor%5D&cauthor=true&cauthor_uid=8150547), [Santarelli A](http://www.ncbi.nlm.nih.gov/pubmed?term=Santarelli A%5BAuthor%5D&cauthor=true&cauthor_uid=8150547), [Biffi M](http://www.ncbi.nlm.nih.gov/pubmed?term=Biffi M%5BAuthor%5D&cauthor=true&cauthor_uid=8150547), [Magnani B](http://www.ncbi.nlm.nih.gov/pubmed?term=Magnani B%5BAuthor%5D&cauthor=true&cauthor_uid=8150547) (1993) Flecainide acetate: concentration-response relationships for antiarrhythmic and electrocardiographic effects. Int J Clin Pharmacol Res 13:211–219
43. Hellestrand KJ, Bexton RS, Nathan AW, Spurrell RA, Camm AJ (1982) Acute electrophysiological effects of flecainide acetate on cardiac conduction and refractoriness in man. Br Heart J 48:140–148
44. Morganroth J, Horowitz LN (1984) Flecainide: its proarrhythmic effect and expected changes on the surface electrocardiogram. Am J Cardiol 53:89B–94B
45. William AJ, McQuinn RL, Walls J (1988) Pharmacokinetics of flecainide acetate in patients with severe renal impairment. Clin Pharmacol Ther 43:449–455
46. Braun J, Kollert JR, Becker JU (1987) Pharmacokinetics of flecainide in patients with mild and moderate renal failure compared with patients with normal renal function. Eur J Clin Pharmacol 31:711–714
47. [Forland SC](http://www.ncbi.nlm.nih.gov/pubmed?term=Forland SC%5BAuthor%5D&cauthor=true&cauthor_uid=3129455), [Burgess E](http://www.ncbi.nlm.nih.gov/pubmed?term=Burgess E%5BAuthor%5D&cauthor=true&cauthor_uid=3129455), [Blair AD](http://www.ncbi.nlm.nih.gov/pubmed?term=Blair AD%5BAuthor%5D&cauthor=true&cauthor_uid=3129455), [Cutler RE](http://www.ncbi.nlm.nih.gov/pubmed?term=Cutler RE%5BAuthor%5D&cauthor=true&cauthor_uid=3129455), [Kvam DC](http://www.ncbi.nlm.nih.gov/pubmed?term=Kvam DC%5BAuthor%5D&cauthor=true&cauthor_uid=3129455), [Weeks CE](http://www.ncbi.nlm.nih.gov/pubmed?term=Weeks CE%5BAuthor%5D&cauthor=true&cauthor_uid=3129455), [Fox JM](http://www.ncbi.nlm.nih.gov/pubmed?term=Fox JM%5BAuthor%5D&cauthor=true&cauthor_uid=3129455), [Conard GJ](http://www.ncbi.nlm.nih.gov/pubmed?term=Conard GJ%5BAuthor%5D&cauthor=true&cauthor_uid=3129455) (1988) Oral flecainide pharmacokinetics in patients with impaired renal function. J Clin Pharmacol 28:259–267
48. Forland SC, Cutler RE, McQuinn RL, Kvam KC, Miller AM, Conard GJ, Parish S (1988) Flecainide pharmacokinetics after multiple dosing in patients with impaired renal function. J Clin Pharmacol 28:727–735
49. Hellestrand KJ, Nathan AW, Bexton RS, Camm AJ (1984) Response of an abnormal sinus node to intravenous flecainide acetate. Pacing Clin Electrophysiol 7:436–439
